# SENP1‐Mediated HSP90ab1 DeSUMOylation in Cardiomyocytes Prevents Myocardial Fibrosis by Paracrine Signaling

**DOI:** 10.1002/advs.202400741

**Published:** 2024-07-11

**Authors:** Zhihao Liu, Xiyun Bian, Lan Li, Li Liu, Chao Feng, Ying Wang, Jingyu Ni, Sheng Li, Dading Lu, Yanxia Li, Chuanrui Ma, Tian Yu, Xiaolin Xiao, Na Xue, Yuxiang Wang, Chunyan Zhang, Xiaofang Ma, Xiumei Gao, Xiaohui Fan, Xiaozhi Liu, Guanwei Fan

**Affiliations:** ^1^ First Teaching Hospital of Tianjin University of Traditional Chinese Medicine National Clinical Research Center for Chinese Medicine Acupuncture and Moxibustion Tianjin 300193 China; ^2^ Tianjin Key Laboratory of Epigenetics for Organ Development of Preterm Infants Tianjin fifth Central Hospital Tianjin 300450 China; ^3^ State Key Laboratory of Component‐Based Chinese Medicine Tianjin 301617 China; ^4^ Central Laboratory Tianjin Fifth Central Hospital Tianjin 300450 China; ^5^ Department of Cardiology Tianjin Chest Hospital Tianjin 300051 China; ^6^ Haihe Laboratory of Modern Chinese Medicine Tianjin 301617 China; ^7^ Pharmaceutical Informatics Institute College of Pharmaceutical Sciences Zhejiang University Hangzhou Zhejiang 310058 China; ^8^ National Key Laboratory of Chinese Medicine Modernization Innovation Center of Yangtze River Delta Zhejiang University Jiaxing 314100 China

**Keywords:** cardiomyocyte, HSP90ab1, myocardial infarction, SENP1

## Abstract

Myocardial infarction (MI) triggers a poor ventricular remodeling response, but the underlying mechanisms remain unclear. Here, the authors show that sentrin‐specific protease 1 (SENP1) is downregulated in post‐MI mice and in patients with severe heart failure. By generating cardiomyocyte‐specific SENP1 knockout and overexpression mice to assess cardiac function and ventricular remodeling responses under physiological and pathological conditions. Increased cardiac fibrosis in the cardiomyocyte‐specific SENP1 deletion mice, associated with increased fibronectin (Fn) expression and secretion in cardiomyocytes, promotes fibroblast activation in response to myocardial injury. Mechanistically, SENP1 deletion in mouse cardiomyocytes increases heat shock protein 90 alpha family class B member 1 (HSP90ab1) SUMOylation with (STAT3) activation and Fn secretion after ventricular remodeling initiated. Overexpression of SENP1 or mutation of the HSP90ab1 Lys72 ameliorates adverse ventricular remodeling and dysfunction after MI. Taken together, this study identifies SENP1 as a positive regulator of cardiac repair and a potential drug target for the treatment of MI. Inhibition of HSP90ab1 SUMOylation stabilizes STAT3 to inhibit the adverse ventricular remodeling response.

## Graphical abstract

SENP1 deficiency in cardiomyocytes induces cardiac fibrosis by promoting Fn transcription and secretion. Extracellular Fn promotes fibroblast activation through integrin‐FAK signaling. HSP90ab1 Lys72 SUMOylation is key site for promoting STAT3‐mediated Fn transcription. Blocking HSP90ab1 SUMOylation at the Lys72 site may be an effective and novel target for reversing adverse ventricular remodeling after myocardial infarction.

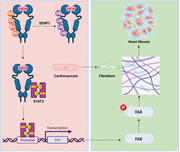

## 1 Introduction

Myocardial infarction (MI), caused by complete or partial occlusion of coronary arteries, is one of the leading causes of death worldwide.^[^ ^1^ ^]^ Inflammatory cells are recruited to remove dead cardiomyocytes, and fibroblasts proliferate and secrete collagen to replace the lost cardiomyocytes. Initially, the repair response is adaptive, but excessive collagen production and scar expansion trigger a maladaptive ventricular remodeling response that promotes the progression of heart failure.^[^ ^2^ ^]^ Therefore, understanding the molecular mechanisms responsible for the progression of maladaptive remodeling in MI is essential for the development of novel targets to reverse pathological remodeling and block the progression of heart failure.

SUMOylation is a reversible posttranslational modification by covalent binding to specific lysine sites on target proteins.^[^ ^3^ ^]^ SUMOylation is critical for the regulation of the cell cycle, cell metabolism, gene transcription, and DNA damage and repair.^[^ ^4^ ^]^ Normally, SUMO proteins bind to substrates through a cascade of E1‐activating enzyme, E2 conjugase, and E3 ligases, while sentrin‐specific proteases (SENPs) are precisely responsible for the removal of SUMO proteins from target proteins.^[^ ^5^ ^]^ The SENPs family consists of six members, SENP1, SENP2, SENP3, SENP5, SENP6, and SENP7. Of all the identified SENPs family, SENP1 was originally reported and has been extensively studied in diseases such as cancer,^[^ ^6^ ^]^ neurodegenerative diseases,^[^ ^7^ ^]^ and cerebral ischemia‐reperfusion.^[^ ^8^ ^]^ However, the role of SENP1 in cardiovascular disease, particularly in cardiac remodeling after MI, remains unclear.

A recent study demonstrated that gene delivery system‐mediated knockdown of SENP1 exacerbates pressure overload‐induced myocardial hypertrophy and interstitial fibrosis, accelerating the progression of heart failure.^[^ ^9^ ^]^ Additionally, heterozygous SENP1 knockout aggravated myocardial ischemia/reperfusion injury by inhibiting the expression of hypoxia‐inducible factor‐1α.^[^ ^10^ ^]^ These results suggest that SENP1 is an important candidate gene for the treatment of cardiovascular disease. However, previous studies have mainly focused on the whole heart to investigate the effects of SENP1, and the role of SENP1 in different types of heart cells has not been reported. Recently, we demonstrated that the regulation of pathological remodeling by SUMO after MI is cell‐specific.^[^ ^11^ ^]^ SENP1 expression in cardiomyocytes and the direct role of SENP1 in pathological remodeling remain unknown. Therefore, it is necessary to evaluate the roles of SENP1 based on specific cell types in normal and pathological cardiac function.

In this study, we not only found that SENP1 was significantly decreased in the post‐infarct hearts but also generated a cardiomyocyte‐specific SENP1 inducible KO mouse, which exhibited markedly increased ischemia‐induced fibrotic response. Unexpectedly, we found that increased myocardial fibrosis in cardiomyocytes‐specific SENP1 deletion mice was associated with enhanced fibronectin (Fn) secretion and upregulation of heat shock protein 90 alpha family class B member 1 (HSP90ab1) in vitro and in vivo. Finally, we demonstrate that inhibiting HSP90ab1 SUMOylation using a gene intervention strategy in cardiomyocytes improves cardiac function after MI as a therapeutic intervention.

## 2 Results

### 2.1 SENP1 Expression Is Downregulated in Cardiomyocytes During Cardiac Remodeling Progression

**Figure 1:**
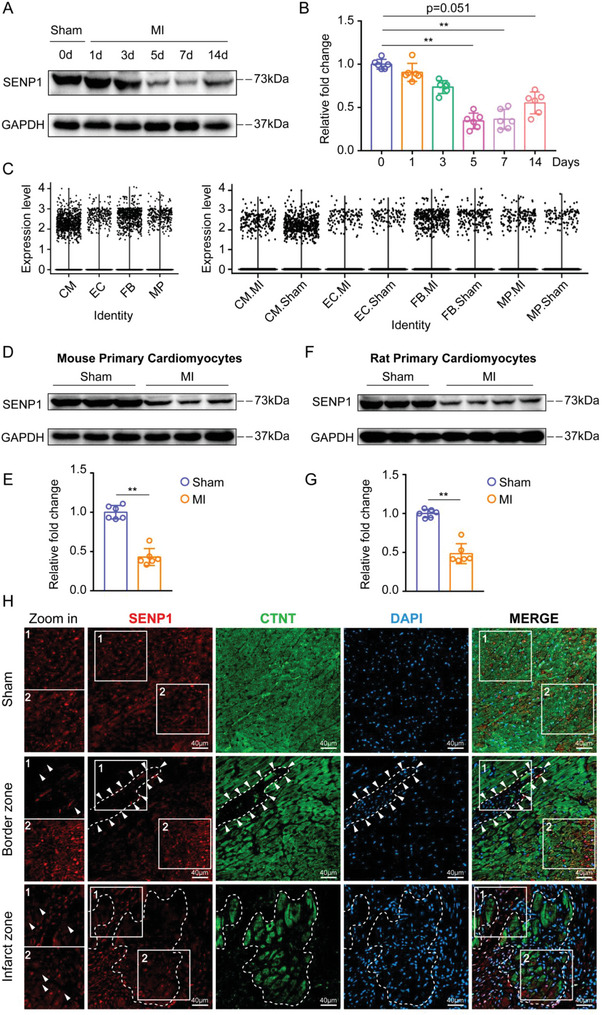
Myocardial injury triggers downregulation of SENP1 in cardiomyocytes. A,B) Time course of mouse cardiac SENP1 expression (non‐infarcted area) in response to myocardial injury with representative immunoblot images (A) and quantitative analyses (B) (*n* = 6). C) Violine plot showing SENP1 scaled expression across the whole cell types in the mouse heart snRNA‐seq data. D,E) Protein levels of SENP1 in primary cardiomyocytes after MI with representative immunoblot images (D) and quantitative analyses (E) in mice (*n* = 6). F,G) Protein levels of SENP1 in primary cardiomyocytes after MI with representative immunoblot images (F) and quantitative analyses (G) in rat (*n* = 6). H) Immunofluorescent staining of SENP1 (red) and CTNT (green) was performed in sham and MI‐injured mouse heart. The dashed area represents the border between the infarct area and the surviving cardiomyocytes. The white arrow indicates the location of cardiomyocytes. Data are shown as mean ± SEM. **p < 0.01, using one‐way ANOVA followed by the Dunn post hoc multiple comparisons test (B), Mann–Whitney *U* test (E,G).

To determine whether SENP1 is involved in the pathological remodeling response, we examined the expression of SENP1 at different time points after MI in mice. In an injury‐time course, we found that SENP1 expression decreases already at 5 days post‐MI, and it is still downregulated at 1 week although to a significantly lower extent (**Figure** **1**A,B). In addition, we collected biopsy samples from four patients subjected to a trans jugular interventricular septal myocardial biopsy and classified the patient's heart function according to NYHA (Table S1, Supporting Information). Immunoblot analysis showed that SENP1 protein levels were also dramatically reduced in the hearts of patients diagnosed with severe heart failure (Figure S1A,B, Supporting Information). To explore the distribution of SENP1 in cardiac tissue, we performed a single‐cell transcriptome analysis in our previously characterized mouse model of MI after coronary artery ligation.^[^ ^11^ ^]^ Unbiased single‐cell transcriptomic analysis revealed a significant reduction in SENP1 levels in cardiomyocytes at 7 days after MI (Figure 1C).

Notably, we found a slight increase in SENP1 expression in heart tissue at day 14 post‐MI (Figure 1A,B). To determine whether it was mediated by cardiomyocytes, we isolated primary cardiomyocytes at different time points and found that SENP1 expression was still low in cardiomyocytes at day 14 after MI (Figure S1C, Supporting Information). Endothelial cell proliferation‐mediated neovascularization is a critical event after resolution of inflammation, especially 2 weeks after MI. We detected the expression of SENP1 in endothelial cells at different time points after MI, and found that SENP1 expression in endothelial cells did not change in the early stage after MI, but increased in the late stage of infarction (Figure S1D, Supporting Information). This may be the possible reason for the slight elevation of SENP1 in the heart tissue at day 14.

The expression level of SENP1 was detected by primary mice ventricular myocytes after MI, a lower SENP1 protein level was observed after myocardial injury than healthy (Figure 1D,E). In addition to mice, the downregulation of SENP1 expression post‐MI was also observed in rats (Figure 1F,G). As shown in Figure S1E (Supporting Information) through G, SENP1 was significantly decreased in neonatal rat ventricular myocytes (NRVMs) in response to ischemic stimulation. To confirm the involvement of SENP1 in cardiomyocyte induction by myocardial injury, we performed immunofluorescence analysis of SENP1 protein levels in cardiac cross‐sections from mice after myocardial injury. Similar to the decrease in SENP1 protein levels and distribution shown in our myocardial injury model, we found reduced SENP1 in the cardiomyocytes surrounding the infarct area (not the distal region) injured by 5 days of MI (Figure 1H). Importantly, we also found reduced SENP1 expression in cardiomyocytes from patients with severe heart failure (Figure S1H, Supporting Information). Collectively, these results showed that SENP1 in myocytes is a critical source in response to myocardial injury and that SENP1 may play a role in cardiac remodeling.

### 2.2 Loss of SENP1 in Cardiomyocytes Induces Cardiac Dysfunction and Myocardial Fibrosis

**Figure 2:**
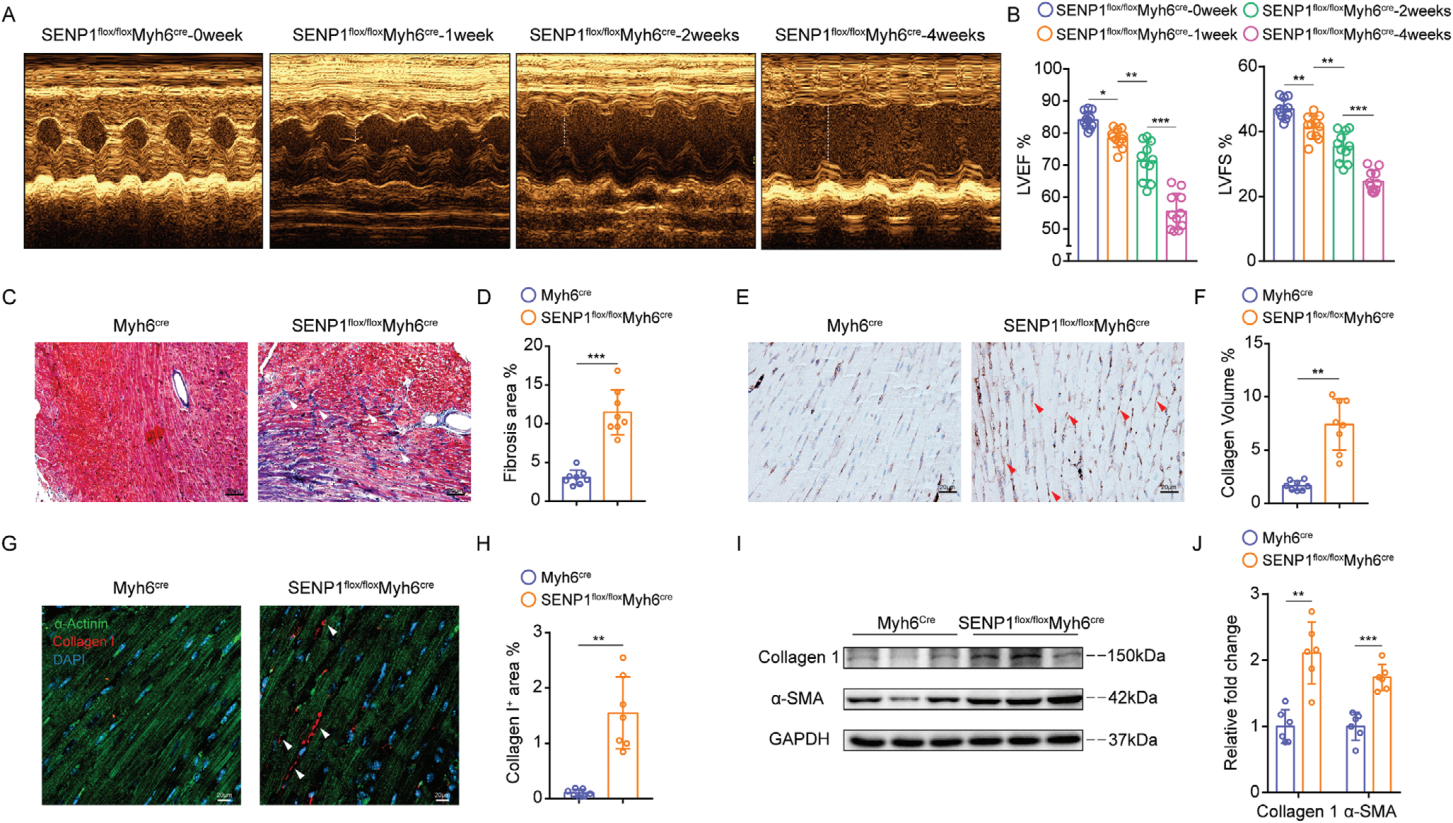
Loss of SENP1 in cardiomyocytes incurs cardiac fibrosis accompanied by deteriorated cardiac function. A) Representative echocardiographic images of SENP1^flox/flox^Myh6^cre^ mice. B) LVEF and LVFS measured by 2D echocardiography on mice (*n* = 11). C,D) Representative images of left ventricle stained with Masson's trichrome staining (C) (scale bar: 50 µm) and quantitative analyses (D) in mice (*n* = 8). E,F) Representative images of left ventricle stained with Collagen I staining (E) (scale bar: 20 µm) and quantitative analyses (F) in mice (*n* = 8). G,H) Immunofluorescence staining for collagen I^+^ (red) and α‐actin^+^ (green) cardiac sections (G) (scale bar: 20 µm) and quantitative analyses (H) (*n* = 7). I,J) Immunoblots for collagen 1 and α‐SMA in SENP1^flox/flox^Myh6^cre^ and Myh6^cre^ mice heart tissues (I) and quantitative analyses (J) (*n* = 6). Data are shown as mean ± SEM. *p < 0.05, **p < 0.01, and ***p < 0.001, using one‐way ANOVA followed by Tukey post hoc multiple comparisons test (B), unpaired *t*‐test with Welch's correction (D,F,H,J).

To assess the role of SENP1, we selectively deleted the SENP1 gene in cardiomyocytes. SENP1^flox/flox^ mice were bred with CRE recombinase expressed under myosin heavy chain 6 (Myh6‐Cre) transgenic mice. Cardiomyocytes and non‐cardiomyocytes were separated by Langendorff perfused isolated mouse hearts and homogenized to detect SENP1 protein levels (Figure S2A, Supporting Information). Eight‐week‐old mice were measured by ultrasound from 1 to 4 weeks after injection of tamoxifen (TAM) 5 days. We found that cardiac performance was significantly reduced in SENP1^flox/flox^Myh6^Cre^ mice compared to controls after tamoxifen induction, including reduced ejection fraction and fractional shortening. The observed cardiac dysfunction persisted for 4 weeks after cardiomyocyte‐specific SENP1 knockout (**Figure** **2**A,B). Histological analysis by Masson's trichrome staining exhibited obvious evidence of interstitial fibrosis within the ventricular myocardium after SENP1 deletion (Figure 2C,D). Significant deposition of collagen was observed in the SENP1^flox/flox^Myh6^Cre^ mice as shown by immunohistochemical staining and immunofluorescence (Figure 2E–H). In addition, we observed significantly increased expression of fibrotic protein, including collagen 1 and α‐smooth muscle actin (α‐SMA), which were significantly elevated in the SENP1^flox/flox^Myh6^Cre^ mice hearts (Figure 2I,J). This deleterious response was not associated with differences in cardiomyocyte hypertrophy or apoptosis following cardiomyocyte‐specific SENP1 loss. (Figure S2B–E, Supporting Information).

To further clarify the functional significance of SENP1 in myocardial fibrosis, we performed tail vein injection of miR30‐based SENP1 shRNA (CtnT promoter) (1 × 10^11^ v g mL^−1^, 100 µL per animal) in mice. The efficacy of the shRNA‐mediated SENP1 defection was evaluated at protein level by immunoblot (Figure S3A, Supporting Information). Echocardiography reveled a progressive reduction in ejection fraction and fractional shortening in SENP1 shRNA‐treated mice (Figure S3B,C, Supporting Information). Histological staining of heart sections showed the expansion of myocardial fibrosis when SENP1 was silenced (Figure S3D–I, Supporting Information). Meanwhile, the expression levels of collagen 1 and α‐SMA were significantly increased in shRNA‐mediated SENP1‐deficient hearts (Figure S3J,K, Supporting Information). These results showed that SENP1 deletion in cardiomyocytes leads to myocardial fibrosis and progressive cardiac dysfunction.

### 2.3 Loss of SENP1 Exacerbates Cardiac Dysfunction and Fibrosis Following Acute Myocardial Injury

**Figure 3:**
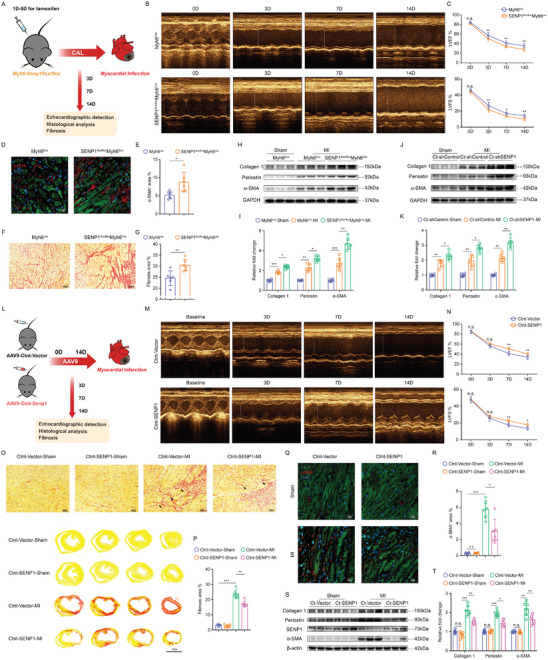
SENP1 deficiency in cardiomyocytes exacerbates fibrosis after myocardial injury and SENP1 overexpression ameliorates adverse ventricular remodeling. A) Schematic of MI model and echocardiography in SENP1^flox/flox^Myh6^cre^ and Myh6^cre^ mice. B) Representative echocardiographic images of SENP1^flox/flox^Myh6^cre^ mice and Myh6^cre^ mice after MI. C) LVEF and LVFS measured by 2‐dimensional echocardiography on mice, at different time points (*n* = 9). D,E) Immunofluorescence staining for α‐SMA^+^ (red) and CTNT^+^ (green) cardiac sections (D) (scale bar: 20 µm) and quantification of α‐SMA^+^ areas per field (E) (*n* = 6). F,G) Representative images of left ventricle stained with Sirius red staining showing degrees of fibrosis (F) (scale bar, 50 µm) and quantitative analyses (G) (*n* = 6). H,I) Immunoblots for collagen 1, periostin, and α‐SMA in SENP1^flox/flox^Myh6^cre^ and Myh6^cre^ mice heart tissues after MI (H) and quantitative analyses (I) (*n* = 6). J, K) Immunoblots for collagen 1, periostin, and α‐SMA in CTNT‐shSENP1 and CTNT‐shControl mice heart tissues after MI (J) and quantitative analyses (K) (*n* = 6). L) Schematic of MI model and echocardiography in CTNT‐SENP1 and CTNT‐Vector mice. M) Representative echocardiographic images of CTNT‐SENP1 mice and CTNT‐Vector mice after MI. N) LVEF and LVFS measured by 2D echocardiography on mice, at different time points (0, 3, 7, and 14 days after MI) (*n* = 7). O,P) Representative images of left ventricle stained with Sirius red staining showing degrees of fibrosis in mice (O) (scale bar: 50 µm in top and 1 cm in bottom) and quantitative analyses (P) (*n* = 6). Q,R) Immunofluorescence staining for α‐SMA^+^ (red) and CTNT^+^ (green) cardiac sections (Q) (scale bar: 20 µm) and quantification of α‐SMA^+^ areas per field (R) (*n* = 6). S,T) Immunoblots for collagen 1, periostin, and α‐SMA in CTNT‐SENP1 and CTNT‐Vector mice heart tissues after MI (S) and quantitative analyses (T) (*n* = 6). Data are shown as mean ± SEM. *p < 0.05, **p < 0.01, and ***p < 0.001, using Two‐way ANOVA with Sidak's multiple comparisons test (C,N), unpaired t test with Welch's correction (E and G), one‐way ANOVA with Tukey's multiple comparisons test (I,K), two‐way ANOVA followed by Sidak post hoc multiple comparisons test (R,P,T).

To test whether SENP1 plays an important role in regulating cardiac remodeling and function after myocardial injury, we measured how the heart responds to cardiac ischemia in the absence of SENP1 in cardiomyocytes by independently assessing cardiac function at 1–14 days post‐MI (**Figure** **3**A). Strikingly, cardiomyocyte specific SENP1‐dedicient mice showed accelerated the damage of cardiac function post‐MI (Figure 3B,C). Since we have shown that deletion of SENP1 in cardiac myocytes establishes profibrotic functions under physiological conditions, we reasoned that SENP1 may be involved in the development of cardiac fibrosis post‐MI and the subsequent development of cardiac dysfunction. A significantly increased fibrosis area and α‐SMA^+^ area around cardiac myocytes were observed in SENP1^flox/flox^Myh6^Cre^ mice compared to Myh6^Cre^ mice after myocardial injury (Figure 3D–G). In addition, western blot analysis revealed significantly increased expression of the fibrotic proteins after SENP1 deletion, including collagen I, periostin, and α‐SMA (Figure 3H,I). Similarly, loss of SENP1 in cardiomyocytes using adenoviral strategies promoted the development of myocardial fibrosis post‐MI (Figure 3J,K). Notably, cardiomyocyte apoptosis was exacerbated in SENP1^flox/flox^Myh6^Cre^ mice heart cross‐sections after MI, compared to the control group (Figure S4, Supporting Information), indicating that silencing of SENP1 in cardiac myocytes exacerbated adverse cardiac remodeling after ischemic injury, defined as severely impaired cardiac function, exacerbated myocardial fibrosis, and increased cardiomyocyte apoptosis.

### 2.4 Genetic Overexpression of SENP1 in Cardiomyocytes Reduces Adverse Ventricular Remodeling Progression

To further determine the critical role of SENP1 in the pathogenesis of pathological myocardial remodeling in vivo, recombinant adeno‐associated virus (serotype 9) vectors carrying mouse SENP1 (accession number NM_144 851) with a c‐TNT promoter (AAV9‐CTNT‐SENP1) were used to investigate the effect of SENP1 overexpression in cardiomyocytes on the degree of myocardial fibrosis after MI (Figure 3L). Non‐invasive echocardiography was used to determine the cardiac function and quantified by percent left ventricular ejection fraction and fractional shortening. Ventricular function was significantly improved in cardiomyocyte‐specific SENP1 overexpression mice as compared to control mice 3–14 days post‐MI (Figure 3M,N). Fibrotic area was significantly reduced in SENP1 overexpression mice as compared to control mice (Figure 3O,P). In addition, less α‐SMA^+^ area was found adjacent to the interstitial space in the boundary regions after overexpression of SENP1 in cardiomyocytes (Figure 3Q,R). Moreover, molecular markers of myocardial fibrosis in vivo were strongly suppressed by SENP1 overexpression at both protein and mRNA levels (Figures 3S,T and S5, Supporting Information). Taken together, loss of SENP1 in cardiomyocytes promoted increased cardiac fibrosis and exacerbated cardiac dysfunction following acute myocardial injury in mice, and overexpression of SENP1 in cardiomyocytes inhibited excessive myocardial fibrosis and adverse ventricular remodeling.

### 2.5 The Paracrine Effect of Cardiomyocytes‐Fibroblasts Is Regulated by SENP1

**Figure 4:**
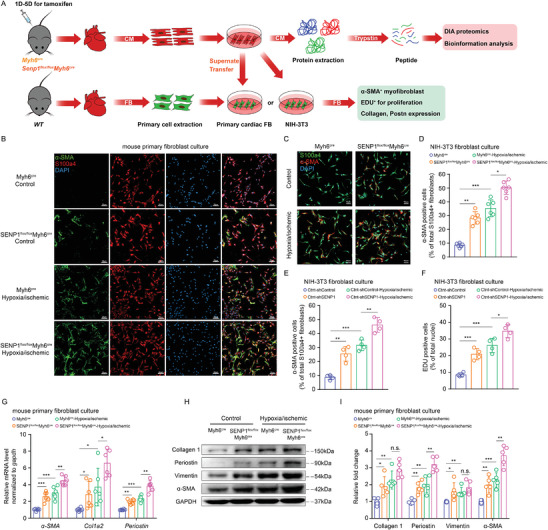
Conditioned medium collected from cardiomyocytes deficient in SENP1 increases the differentiation and proliferation of fibroblasts. A) Schematic representation of the in vitro assay for paracrine signaling between primary cardiac myocytes and primary fibroblasts or NIH‐3T3. Conditioned medium derived from SENP1^flox/flox^Myh6^cre^ and Myh6^cre^ mice primary cardiomyocytes for fibroblast stimulation and primary cardiomyocytes for DIA proteome sequencing. B) Immunofluorescence staining for α‐SMA^+^ (green) and S100a4^+^ (red) primary fibroblasts (scale bar: 20 µm). C,D) Immunofluorescence staining for S100a4^+^ (green) and α‐SMA^+^ (red) NIH‐3T3 fibroblasts (C) (scale bar: 50 µm) and quantification of S100a4‐α‐SMA double positive cells per field (D) (*n* = 6). E,F) Quantification of S100a4‐α‐SMA double positive cells per field (E) and EDU^+^ cells per field (F) of NIH‐3T3 fibroblasts (*n = 6*). G, *α‐SMA*, *Col1a2*, and *periostin* mRNA expression in mouse primary fibroblasts stimulated by conditioned medium (*n* = 6). H,I) Immunoblots for collagen 1, periostin, vimentin, and α‐SMA in mouse primary fibroblasts after conditioned medium stimulation (H) and quantitative analyses (I) (*n* = 5). Data are shown as mean ± SEM. *p < 0.05, **p < 0.01, and ***p<0.001, using two‐way ANOVA followed by Sidak post hoc multiple comparisons test (D–G,I).

The observation that SENP1 knockout mice have caused cardiac fibrosis and functional decompensation under physiological conditions. However, we found no apoptotic response in cardiomyocytes, which prompted us to test whether cardiomyocytes, upon deletion of SENP1 induction, would signal directly to cardiac fibroblasts. To address these questions, we isolated primary cardiomyocytes from Myh6^Cre^ and SENP1^flox/flox^Myh6^Cre^ mice and performed intervention under normal and ischemic (hypoxia and serum starvation) conditions. We then transferred mouse primary fibroblasts or embryonic fibroblast cell lines (NIH‐3T3) into cardiomyocyte media (**Figure** **4**A). Immunofluorescence analysis showed that conditioned media from SENP1‐deleted myocytes promoted the number of activated (α‐SMA^+^) fibroblasts (Figure 4B‐F and Figure S6A–C, Supporting Information). To further test this hypothesis, we cultured cardiac fibroblasts with the conditioned medium from cardiomyocytes infected with AAV‐shSENP1. As expected, treatment of cardiac fibroblasts with the conditioned medium for 24 h significantly increased fibroblast proliferation and myofibroblast differentiation compared to shControl medium, and this effect was enhanced by pre‐ischemia of cardiomyocytes (Figure S6D,E, Supporting Information). Moreover, molecular markers of myocardial fibrosis in mouse primary fibroblasts were strongly promoted by SENP1‐deficient primary myocyte medium at both mRNA and protein levels (Figure 4G–I). These results demonstrate that SENP1 deletion in cardiomyocytes accelerates fibroblast proliferation and activation through a paracrine effect.

### 2.6 Deletion of SENP1 Promotes Fn Expression and Secretion in Cardiomyocytes

**Figure 5:**
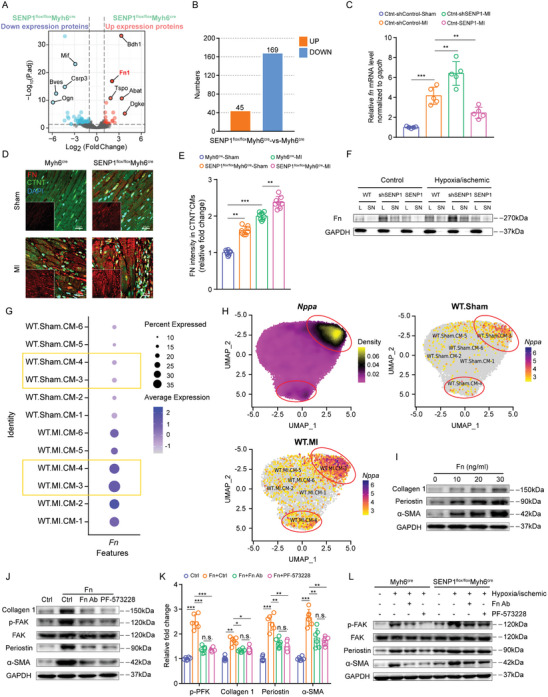
Loss of SENP1 in cardiomyocytes promotes Fn secretion and activation of FAK signaling in fibroblasts. A) Volcano plot of differentially expressed proteins in SENP1^flox/flox^Myh6^cre^ primary cardiomyocytes compared with those in Myh6^cre^ primary cardiomyocytes. B) Number of differential proteins in SENP1^flox/flox^Muh6^cre^ primary cardiomyocytes compared with Myh6^cre^ primary cardiomyocytes. C) *Fn* mRNA expression in mouse primary cardiomyocytes (*n* = 5). D,E) Immunofluorescence staining for Fn (red) and CTNT (green) cardiac sections (D) (scale bar: 20 µm) and quantification of Fn^+^ areas per field (E) (*n* = 8). F) Immunoblots for Fn in mouse primary cardiomyocytes and conditioned medium from adenovirus‐transfected mice. G) Dot plot showing Fn expression for each cardiomyocyte subclusters. H) UMAP visualization of clustering revealed Nppa expression for each cardiomyocyte subclusters. I) Immunoblots for collagen 1, periostin, and α‐SMA in primary fibroblasts cultured in the absence (control) or presence of Fn (at indicated concentrations). J,K) Immunoblots for collagen 1, periostin, and α‐SMA in primary fibroblasts cultured in the presence of Fn neutralizing antibody and PF‐573228 (inhibitors of FAK signaling) (J) and quantitative analyses (K) (*n* = 6). L) Immunoblots for primary fibroblasts cultured with SENP1‐deleted myocytes conditioned media in the presence of Fn neutralizing antibody and PF‐573228. Data are shown as mean ± SEM. *p < 0.05, **p < 0.01, and ***p < 0.001, using one‐way ANOVA with Tukey's multiple comparisons test (C,K), two‐way ANOVA followed by Sidak post hoc multiple comparisons test (E).

To identify putative paracrine factors, primary cardiomyocytes from Myh6^Cre^ and SENP1^flox/flox^Myh6^Cre^ mice were subjected to DIA assay and bioinformatics analysis. We found that the secreted protein fibronectin (Fn) was significantly increased in SENP1‐deficient cardiomyocytes (**Figure** **5**A,B and Tables S2 and S3, Supporting Information). Fn is a highly conserved protein with multiple biological roles in development, cell growth and differentiation, adhesion, migration, and wound healing, mainly through integrin‐mediated signaling.^[^ ^12^ ^]^ Moreover, cardiac myocytes in shSENP1 group had higher Fn mRNA and protein levels than those in the shControl group (Figure 5C–E). We then used immunoblotting to detect Fn in the cell lysate and conditioned medium, and found that the absence of SENP1 promoted the expression and release of Fn in primary cardiac myocytes, and this effect was enhanced by pre‐ischemia of the cardiac myocytes. However, Fn expression was significantly reduced in myocytes overexpressing SENP1 compared to ischemic myocytes (Figure 5F). To investigate the expression status of Fn in pre‐ and post‐infarction mouse hearts, we then analyzed single‐cell RNA sequencing data from cardiomyocytes alone. Consistent with the data in Figure 5C, *Fn* expression was increased in cardiomyocytes subsets after MI, particularly in the CM‐3 cluster characterized by *Nppa*, which responds to myocardial injury (Figure 5G,H). Recombinant Fn was administered to primary mouse fibroblasts, and the pro‐fibrosis effects of Fn were found to be concentration dependent (Figure 5I). Given that focal adhesion kinases (FAKs) are the most prominent proteins involved in the ECM‐integrin signaling pathways critical for fibroblast proliferation/activation,^[^ ^13^ ^]^ a neutralizing antibody of ITGB1 and ATN‐161(Small peptide antagonist of integrin α5β1) also blocks Fn‐induced activation of FAK in fibroblasts (Figure S7A, Supporting Information). We used a neutralizing antibody (Fn Ab) and a selective FAK inhibitor (PF‐573228) to detect the activation of FAK signaling. Importantly, application of Fn Ab and PF‐573228 in the medium reduced the abundance of phospho(p)‐FAK, collagen I, periostin, and α‐SMA in fibroblasts caused by the absence of SENP1 in cardiomyocytes (Figure 5J–L). Previous studies have shown that factors such as platelet‐derived growth factor and vascular endothelial can activate FAK signaling through the autophosphorylation of PDGFRB^[^ ^14^ ^]^ and VEGFR2.^[^ ^15^ ^]^ To determine whether Fn mediates FAK activation by regulating PDGFRB and VEGFR2 signaling, we examined the phosphorylation of PDGFR2 and VEGFR2 after administration of Fn and the ablative antibody Fn‐ab to fibroblasts. We found that Fn did not affect the expression and phosphorylation activation of PDGFRB and VEGFR2 (Figure S7B, Supporting Information). Together, Fn was released from damaged cardiomyocytes and mediated fibroblast proliferation and activation through FAK signaling, and the loss of SENP1 exacerbated the production and release of Fn in cardiomyocytes.

### 2.7 SENP1 Inhibits STAT3‐Mediated Fn Transcription in Cardiomyocytes

**Figure 6:**
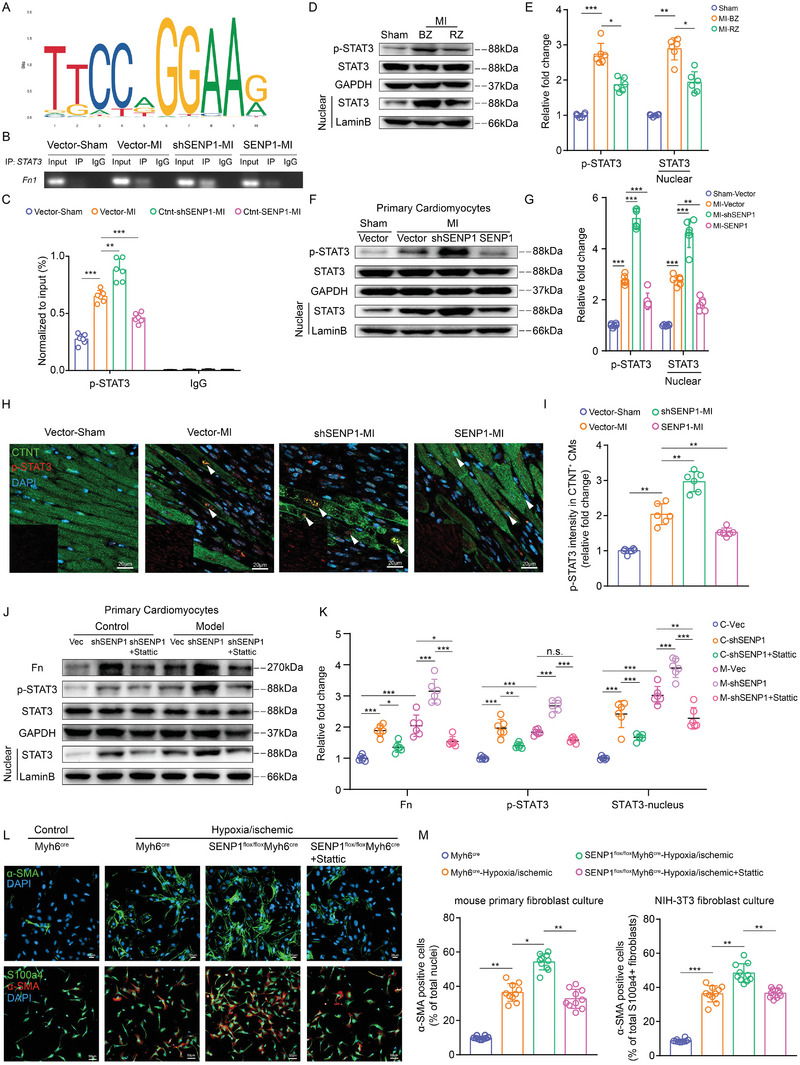
SENP1 represses STAT3‐mediated transcription of Fn in cardiomyocytes. A) Schematic representation of conserved STAT3 binding sequences identified in the promoter regions of *Fn* using JASPAR. B,C) The DNA fragment derived from mouse primary cardiomyocytes corresponding to the Fn promoter enriched by STAT3 binding was evaluated by agarose gel electrophoresis (B) or real‐time quantitative PCR (C) (*n* = 6). D,E) Immunoblots for STAT3 and p‐STAT3 in the border zone (BZ) and remote zone (RZ) of the heart tissue (D) and quantitative analyses (E) (*n* = 6). F,G) Immunoblots for STAT3 and p‐STAT3 in the cytoplasm and nucleus of cardiomyocytes from sham and MI‐injured (AAV‐mediated CTNT‐Vector, CTNT‐shSENP1, and CTNT‐SENP1) mouse heart (F) and quantitative analyses (G) (*n* = 6). H,I) Immunofluorescence staining for p‐STAT3 (red) and CTNT (green) cardiac sections (H) (scale bar: 20 µm) and quantification of p‐STAT3^+^ areas in CTNT^+^ cardiomyocyte nuclei per field (I) (*n* = 6). J,K) Immunoblots for Fn, STAT3 and p‐STAT3 of primary cardiomyocytes in control and hypoxia/ischemic conditions (J) and quantitative analyses (K) (*n* = 6). L) Immunofluorescence staining for α‐SMA^+^ (red) primary fibroblasts, and S100a4^+^ (green) and α‐SMA^+^ (red) NIH‐3T3 fibroblasts (scale bar: 20 µm on top and 50 µm on bottom). M) Quantification of α‐SMA^+^ cells per field and S100a4‐α‐SMA double positive cells per field (*n* = 10). Data are shown as mean ± SEM. *p < 0.05, **p < 0.01, and ***p < 0.001, using one‐way ANOVA with Tukey's multiple comparisons test (C,G,I,K,M), one‐way ANOVA followed by the Dunn post hoc multiple comparisons test (E).

Given that Fn is altered at the transcriptional level following injury and SENP1 deletion in cardiomyocytes, we used the GTRD database (http://gtrd20‐06.biouml.org/), the hTFtarget database (http://bioinfo.life.hust.edu.cn/hTFtarget#!/), and the HumanTFDB database (binding score >21) (http://bioinfo.life.hust.edu.cn/HumanTFDB#!/) to screen for transcription factors (TFs) at the *Fn* promoter and identified 14 TFs, followed by sequence alignment using JASPAR (https://jaspar.genereg.net/) to verify motifs. We found that forkhead box A2, interferon regulatory factor 1, serum response factor, signal transducer and activator of transcription 3 (STAT3), and sp1 transcription factor have motifs that bind to the *Fn* promoter, with STAT3 having the strongest site‐specific binding (Figure S8A and Table S4, Supporting Information). In addition, published ChIP‐seq data reveled 19 STAT3 binding sites within the 1kb region of the *Fn* TSS, suggesting that the potential for direct transcriptional regulation of Fn by STAT3.^[^ ^16^ ^]^ Conserved binding sequences in the promoter regions of *Fn* were identified using JASPAR (https://jaspar.genereg.net/) (**Figure** **6**A). CHIP analysis showed that STAT3 binds to the *Fn* promoter after myocardial injury, and this was enhanced by SENP1 deletion. However, overexpression of SENP1 in myocytes inhibited the occupancy of STAT3 in the *Fn* promoter region (Figure 6B, C). Considering the reduced expression of SENP1 in cardiomyocytes in the infarct border zone, western blot analysis of the infarct border zone and distal zone of heart tissue showed that STAT3 Tyr‐705 phosphorylation and nuclear STAT3 levels were significantly increased after MI, especially in the border zone (Figure 6D,E). Western blot and immunofluorescence assays showed that myocardial injury increased STAT3 phosphorylation and nuclear translocation. As expected, loss of SENP1 strongly increased STAT3 phosphorylation and nuclear translocation in cardiac myocytes, which was blocked by overexpression of SENP1 (Figure 6F–I). To investigate the role of STAT3 in SENP1‐mediated Fn transcription, we added Stattic, an inhibitor of STAT3, showed that Stattic significantly inhibited SENP1 deletion‐mediated Fn expression (Figure 6J,K). And overexpression of STAT3 in cardiomyocytes significantly reversed the inhibitory effect of SENP1 on Fn (Figure S8B, Supporting Information). We further investigated whether STAT3 inhibition attenuated the intense pro‐fibrosis mediated by SENP1 deletion. Immunofluorescence analysis revealed a significantly reduced number of activated fibroblasts in SENP1 deletion primary myocyte medium after application of Stattic compared to SENP1flox primary myocyte medium (Figure 6L,M). These results demonstrate that SENP1 inhibits STAT3‐mediated Fn transcription in damaged cardiomyocytes.

### 2.8 SENP1 Interacts with HSP90ab1 and Regulates Its Stability

Recent studies have shown that overexpression of SUMO2 promotes STAT3 phosphorylation,^[^ ^17^ ^]^ and another study found that SUMOylation of the STAT3 Lys451 site promotes binding to the phosphatase TC45.^[^ ^18^ ^]^ To determine whether SENP1 directly regulates the sustained activation of STAT3, we combined the STAT3 Lys451 mutation on a SENP1‐deficient background and we found that mutation of the SUMOylation site of STAT3 failed to inhibit the sustained phosphorylation of STAT3 induced by SENP1 deletion (Figure S8C,D, Supporting Information). Furthermore, mutation of STAT3 Lys451 did not affect the binding of STAT3 to the *Fn* promoter (Figure S8E, Supporting Information), suggesting that SUMOylation at the STAT3 Lys451 site failed to affect STAT3‐mediated transcription of Fn.

**Figure 7:**
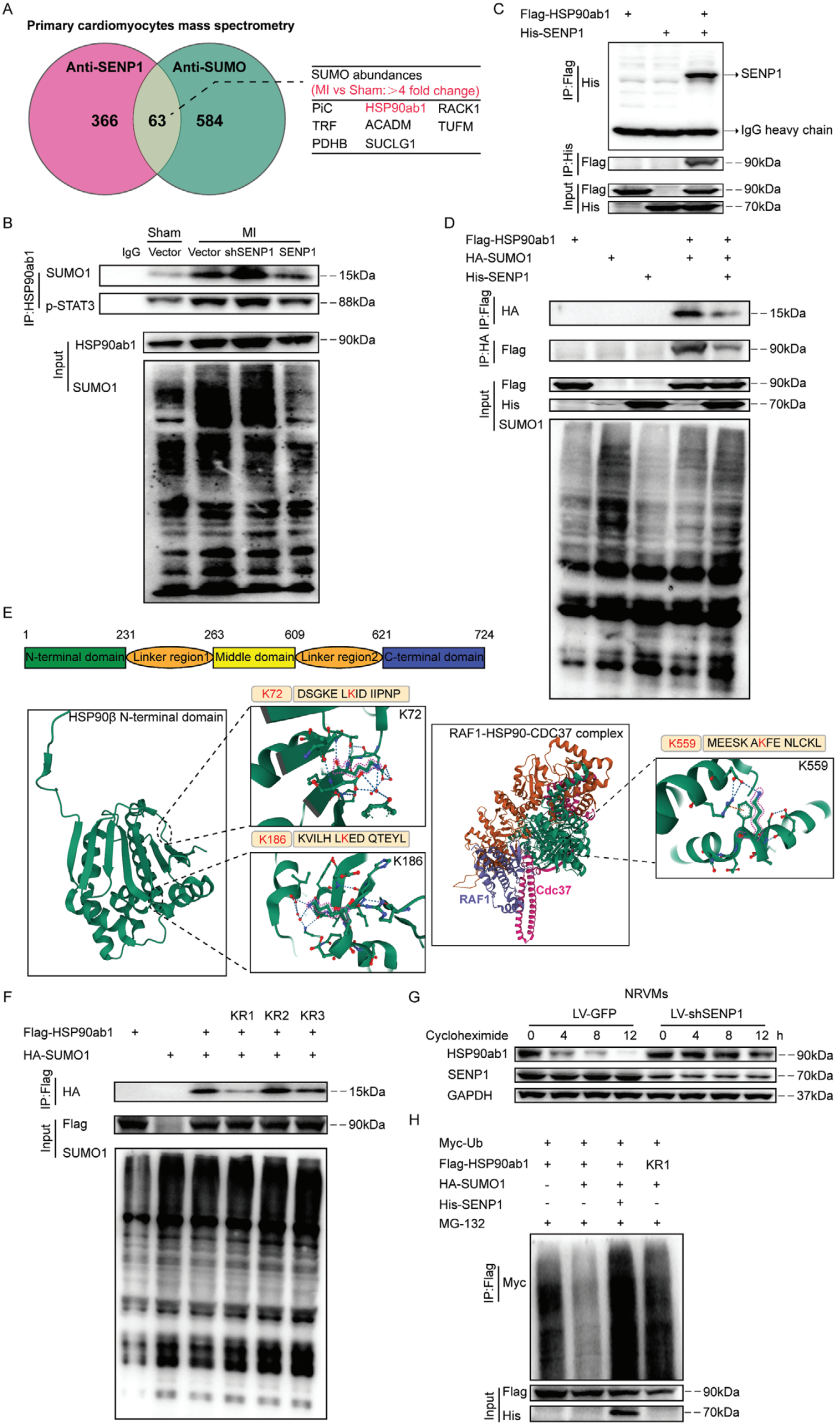
SENP1 promotes HSP90ab1 proteasome degradation by inhibiting SUMOylation at the K72 site of HSP90ab1. A) Mass spectrometry analysis of peptides pulled down using anti‐SENP1 or anti‐SUMO1 antibody in primary cardiomyocytes. B) Coimmunoprecipitation (Co‐IP) assays of the interaction between HSP90ab1 and SUMO1 or p‐STAT3 in primary cardiomyocytes from mice treated with the indicated conditions. C,D) Co‐IP assays of the interaction between HSP90ab1 and SENP1 (C), HSP90ab1 and SENP1 or SUMO1 (D) in HEK293T cells transfected with the indicated plasmids. E) The domain structure of HSP90ab1 and the K72, K186, and K559 sites in HSP90ab1 are shown (Image from RCSB PDB public database. Left: PDB ID: 6N8W; Right: 7Z37). F) Co‐IP assays using wild‐type HSP90ab1 and K72(KR1), K186(KR2), or K559(KR3) mutant HSP90ab1. G) Immunoblotting analysis of HSP90ab1 in neonatal rat ventricular myocytes (NRVMs) applied with cycloheximide (CHX, 50 µm) for the indicated time points. H) Results of ubiquitination assays confirming the ubiquitination of HSP90ab1 after transfected with the indicated plasmids for 24 h and treated with MG132 (50 µm) for 6 h in HEK293T cells.

To investigate the molecular mechanisms by which SENP1 regulates STAT3 in cardiomyocytes, we performed immunoprecipitation‐mass spectrometry using anti‐SENP1 and anti‐SUMO in primary cardiomyocytes. We identified 63 proteins that bind SENP1 and SUMO. Considering that the reduction of SENP1 in cardiomyocytes is critical for the response to myocardial injury, we screened for proteins with high SUMO‐binding abundance (>4‐fold change) in cardiomyocytes after myocardial injury, including PiC, TRF, PDHB, HSP90ab1, ACADM1, SUCLG1, RACK1, and TUFM. Among the interacting SENP1 and SUMO proteins, we identified HSP90ab1 (≈7‐fold change) (**Figure** **7**A and Table S5, Supporting Information), which is a key regulator of protein homeostasis under both physiological and pathological conditions in adult cardiomyocytes and binds to STAT3 as a molecular chaperone.^[^ ^19^ ^]^ Given the accumulation of STAT3 in the nucleus upon SENP1 deletion, this interaction is particularly interesting. Coimmunoprecipitation analysis showed that endogenous HSP90ab1‐SUMO1 and HSP90ab1‐pSTAT3 interactions were significantly increased in the shSENP1 group compared to controls in cardiomyocytes, which was reversed by overexpression of SENP1 (Figure 7B). The interaction between HSP90ab1 and SENP1 was then confirmed by coimmunoprecipitation of overexpressed Flag‐HSP90ab1 and His‐SENP1 in HEK‐293T (human embryonic kidney) cells. Indeed, we found that SUMO1 could directly interact with HSP90ab1 and that this interaction was reduced after SENP1 overexpression (Figure 7C,D). We predicted three SUMOylation sites of HSP90ab1 at K72, K186, and K559 using JASSA (http://www.jassa.fr/index.php?m=jassa) (Figure 7E). Targeting analysis of the mutation of the three lysine residues to arginine on HSP90ab1 (HSP90ab1 K72R, HSP90ab1 K186R, or HSP90ab1 K559R) showed that HSP90ab1 K72 was the most critical SUMOylation site (Figure 7F). To determine the effect of SENP1 on HSP90ab1 in cardiomyocytes, we restricted the expression of SENP1 in NRVMs. We found that SENP1 inhibition did not affect the mRNA level of HSP90ab1 (Figure S9A, Supporting Information), and further analysis of single‐cell sequencing data showed that damaged cardiomyocyte cluster did not alter HSP90ab1 gene expression (Figure S9B, Supporting Information). However, deletion of SENP1 in NRVMs increased HSP90ab1 protein expression (Figure S9C,D, Supporting Information). SENP1 deletion inhibited cycloheximide‐induced HSP90ab1 degradation (Figure 7G). We therefore hypothesized that SENP1 might promote HSP90ab1 ubiquitination by blocking HSP90ab1 SUMOylation. To test this hypothesis, we further examined the degree of ubiquitination of HSP90ab1. Overexpression of SUMO1 reduced the ubiquitination level of HSP90ab1, which was reversed by mutations at the K72 site of HSP90ab1 (Figure 7H).

### 2.9 Blocking HSP90 Lys^72^ SUMOylation Restored Cardioprotective Signaling and Reversing Pathological Remodeling

**Figure 8:**
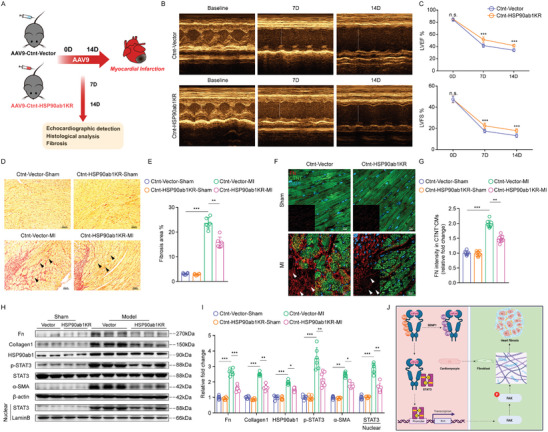
Cardiomyocyte‐specific HSP90ab1 K72R gene therapy attenuates MI–induced cardiac dysfunction and fibrosis. A) Schematic of MI model and echocardiography in CTNT‐Vector mice and CTNT‐HSP90ab1KR mice. B) Representative echocardiographic images of CTNT‐Vector mice and CTNT‐HSP90ab1KR mice after MI. C) LVEF and LVFS measured by 2‐dimensional echocardiography on mice, at different time points (0, 7, and 14 days after MI) (*n* = 8). D,E) Representative images of left ventricle stained with Sirius red staining showing degrees of fibrosis (D) in mice (scale bar: 50 µm) and quantitative analyses (E) (*n* = 6). F,G) Immunofluorescence staining for Fn^+^ (red) and CTNT^+^ (green) cardiac sections (F) (scale bar: 20 µm) and quantitative analyses of Fn^+^ areas in CTNT^+^ cardiomyocytes per field (G) (*n* = 8). H,I) Immunoblots for Fn, collagen 1, HSP90ab1, STAT3, p‐STAT3 and α‐SMA, in primary cardiomyocytes from mice (H) and quantitative analyses (I) (*n* = 6). J) Graphic illustration. In cardiomyocytes, HSP90ab1 is SUMOylated at lysine 72 and resists ubiquitination after MI, followed by STAT3‐mediated activation of Fn transcription and secretion, thereby promoting FKA signaling‐dependent fibroblast proliferation and activation. Cardiomyocyte‐specific SENP1 overexpression or HSP90ab1K72 mutation reduces its stability and exerts cardioprotective effect after MI (Created with BioRender.com. Agreement number: NE266NFNBL). Data are shown as mean ± SEM. *p < 0.05, **p < 0.01, and ***p < 0.001, using two‐way ANOVA followed by Sidak post hoc multiple comparisons test (C,E,J,I).

Because SENP1 was reduced after MI and activation of SENP1 can inhibit excessive STAT3 activation and levels in cardiomyocyte induced by HSP90ab1 over‐SUMOylation, we then examined whether the SUMOylation site of HSP90ab1 could be used to treat MI‐induced cardiac dysfunction. Recombinant adeno‐associated virus (serotype 9) vectors carrying a mouse sumo site‐mut of HSP90ab1 (HSP90ab1K72R) (accession number NM_0 08302.3) with a c‐TNT promoter (AAV9‐CTNT‐HSP90ab1KR) were used to investigate the effect of SUMOylation at HSP90ab1 K72 on cardiac function after MI (**Figures** **8**A and S10A, Supporting Information). Mice carrying HSP90ab1K72R showed preserved cardiac function versus deteriorating cardiac function in the vector group (Figure 8B,C). Mutation at the SUMOylation site of HSP90 significantly reduced fibrosis (Figure 8D,E) and suppressed Fn expression in cardiomyocytes surrounding the infarct induced by ischemic injury (Figure 8F,G). Furthermore, SUMOylation site depletion of HSP90ab1 abolished the effect of SENP1 deficiency on the phosphorylation and accumulation of STAT3 in the nucleus of cardiomyocytes (Figure 8H,I). In addition, mutations at the K72 site of HSP90ab1 in cardiomyocytes inhibited the strong pro‐fibrotic effect mediated by SENP1 deletion (Figure S10B,C, Supporting Information). Taken together, these data demonstrate that HSP90ab1 serves as an essential target of SENP1 in myocardial ischemia and that targeting HSP90ab1 SUMOylation may provide therapeutic strategies for pathological cardiac remodeling (Figure 8J).

## 3 Discussion

Pathological ventricular remodeling is a major component of ischemic heart disease. An incomplete understanding of the molecular mechanisms of cardiac fibrosis after MI has hindered the development of effective strategies to reverse heart failure. In this study, we report, for the first time, that SENP1 is decreased develops 5–7 days after MI, a critical time window when the adaptive/pathological remodeling transition occurs, and it is a key endogenous regulator of myocardial homeostasis in the physiological and pathological heart. Yang et al discovered a novel role of SENP1 in the pathogenesis of cardiac hypertrophy.^[^ ^9^ ^]^ By transducing murine hearts with AAV9 vectors carrying shSENP1, the authors found that the absence of cardiac global SENP1 exacerbated pressure overload‐induced cardiac dysfunction. These results suggest that SENP1 is critical for the ability of cardiomyocytes to withstand extra work. Similarly, our results show that SENP1 protected cardiomyocytes from the effects of myocardial ischemia, as the absence of SENP1 in cardiomyocytes exacerbates cell damage. Specifically, conditionally deleted SENP1 in adult cardiomyocytes induces impaired cardiac function, and exacerbates the adverse cardiac remodeling response after MI. Conversely, increasing SENP1 abundance in cardiomyocytes inhibits Fn secretion and fibroblast activation, suggesting that SENP1 is required for maintaining cardiomyocyte homeostasis and is protective against stresses altering cardiomyocyte proteostasis.

In this study, we made three novel observations. First, we observe that SENP1 deficiency in cardiomyocytes promotes Fn secretion and induces progressive myocardial fibrotic responses. Expression of the ECM protein Fn maintains at high levels during early postnatal development and declines in adulthood. After a pathological injury such as myocardial ischemia or pressure overload, Fn is re‐induced in the myocardium and exerts paracrine effects to mediate the ventricular remodeling response. Additionally, epicardial‐secreted Fn mediates epicardial‐cardiomyocyte crosstalk and drives the maturation of hPSC‐derived cardiomyocytes.^[^ ^20^ ^]^ Previous studies have shown that Fn stimulates collagen production in renal epithelial cells in a dose‐dependent manner,^[^ ^21^ ^]^ and similarly, our study shows that Fn administration promotes fibroblast activation by mediating the activation of integrin‐FAK signaling. Therefore, sustained Fn production and deposition in the extracellular matrix increases cardiac stiffness while continuously stimulating fibroblast‐to‐myofibroblast conversion and increased collagen secretion, leading to accelerated deterioration of adverse remodeling after MI. Genetic deletion^[^ ^22^ ^]^ or pharmacological suppression^[^ ^13^ ^]^ of Fn delays myocardial hypertrophy in pressure overload or ischemic cardiac remodeling, thereby improving short‐term survival. Although Fn has been extensively studied in the autocrine regulation of fibroblasts,^[^ ^23^ ^]^ the regulatory mechanisms and paracrine roles of Fn in cardiac myocytes have been rarely been reported. In our study, we found that cardiomyocytes around the infarct area significantly expressed and released Fn to mediate cardiomyocyte‐fibroblast communication, which was also reflected in our single‐cell sequencing dataset. We determine that Fn was highly expressed in cardiomyocyte subsets after MI, especially in the Nppa^+^ cluster, and thus, the stress cardiomyocytes induced by ischemic injury are responsible for the release of Fn. In fact, injured cardiomyocytes regulate surrounding cells through a variety of factors, for example, hypoxia‐induced mitogenic factor ‐mediated IL‐6 secretion from cardiomyocytes promotes fibroblast activation via mitogen‐activated protein kinase and calcium‐calmodulin‐dependent protein kinase II signaling.^[^ ^24^ ^]^ Thymosin β4 and prothymosin α are transcriptionally regulated by zinc finger E‐Box binding homeobox 2 in stressed cardiomyocytes and subsequently secreted to mediate cross‐talk between cardiomyocytes and endothelial cells.^[^ ^25^ ^]^ Although our study shows that Fn secreted by cardiomyocytes regulates myocardial fibrosis 5–7 days after MI, it is worth noting that myocardial injury is a phased process, and the paracrine effect of cardiomyocytes on inflammatory cells (including neutrophil infiltration, macrophage transformation) at the initial stage also needs to be determined in the future.

Further analysis reveals that activation and high nuclear expression of STA3 in cardiomyocytes after myocardial ischemia is responsible for high transcript levels of Fn, which is more obvious after cardiomyocyte‐specific deletion of SENP1. STAT3 is a signaling molecule capable of transporting extracellular and cytoplasmic stimuli to the nucleus in response to a variety of pathological stress responses. Recent studies have extensively demonstrated the role of STAT3 in cardiac hypertrophy,^[^ ^26^ ^]^ ischemia/reperfusion injury,^[^ ^27^ ^]^ diabetic cardiomyopathy,^[^ ^28^ ^]^ and heart failure.^[^ ^29^ ^]^ Sustained Tyr705 site phosphorylation‐mediated STAT3 activation and nuclear translocation is associated with poor prognosis in cardiac hypertrophy and heart failure. It has been shown that SUMOylation at the Lys451 site promotes STAT3 binding to the phosphatase TC45 and affects nuclear translocation in head and neck cancer cell lines.^[^ ^18^ ^]^ In contrast, we found that mutation at the Lys451 site failed to inhibit the strong phosphorylation of STAT3‐Tyr705 induced by SENP1 deletion, suggesting that the reduction of SENP1 induced by ischemic injury mediates STAT3 activation and nuclear translocation is not dependent on SUMO molecule‐mediated binding of STAT3 to the phosphatase TC45, and that regulation of STAT3 by SENP1 is not solely dependent on de‐SUMOylation.

Second, we demonstrate that SENP1‐dependent de‐SUMOylation of HSP90ab1 followed by ubiquitin‐mediated degradation inhibits continued activation of STAT3. We identify HSP90ab1 of the HSP90 family as a key molecule in the SNEP1‐mediated regulation of STAT3 through protein database and mass spectrometry analysis. Hsp90 interacts primarily with proteins involved in transcriptional regulation and signaling transduction pathways, such as protein kinases, ribonucleoproteins, and TFS. A study shows that XL888 (an HSP90 inhibitor without affecting other kinases) inhibits the formation of HSP90‐STAT3 complexes, thereby reducing the expression levels of STAT3 and p‐STAT3 in early‐stage hepatocellular carcinoma.^[^ ^30^ ^]^ In addition, luteolin blocks the association of HSP90 with STAT and mediates the degradation of phosphorylated STAT3 (Tyr705 and Ser727) via a proteasome dependent pathway.^[^ ^31^ ^]^ A recent study shows that the N‐terminal ATP‐binding pocket of HSP90 is dispensable, the compound NCT‐80 degrades STAT3 by binding to the ATP‐binding pocket of HSP90 and thereby disrupting the interaction of HSP90 with STAT3.^[^ ^32^ ^]^ The highly conserved N‐terminal domain of HSP90ab1 primarily provides sites for ATP binding. In particular, numerous studies have shown that the N‐terminal domain of HSP90 provides binding sites for co‐chaperones, including CDC37,^[^ ^33^ ^]^ AHA1,^[^ ^34^ ^]^ and p23/Sba1,^[^ ^35^ ^]^ thereby regulating HSP90 activity or the ability to bind to other ligand proteins. Therefore, the amino acid sites in the N‐terminal domain of HSP90 are critical for the function of HSP90. Indeed, the binding of HSP90 to SUMO1 has been demonstrated in plasma cells^[^ ^36^ ^]^ and neurons.^[^ ^37^ ^]^ However, the regulatory function of SUMO modifications on HSP90 is unclear. We predicted three SUMOylation sites (Lys72, Lys186, and Lys559.) for HSP90ab1. Of these, the Lys72 site in the N‐terminal domain and Lys559 in the middle domain responded to the SUMOylation of HSP90ab1, and mutation of the Lys 72 site to Arg maximally eliminated the degree of SUMOylation of HSP90ab1. We found that SUMOylation at the Lys 72 site resists ubiquitination modification and increases the stability of HSP90ab1. Our identification of this critical lysine site on HSP90ab1 not only advances our understanding of SENP1‐HSP90ab1‐STAT3 axis‐mediated signaling, but may also aid in the development of future precision therapies.

Finally, and most importantly, virus‐mediated in vivo injection of a cardiomyocyte‐specific mutation at the HSP90ab1K72 site inhibits the extent of myocardial fibrosis and improves cardiac function after MI. In fact, in addition to SUMOylation, HSP90 is regulated by a variety of post‐translational modifications and is involved in cardiac disease processes. For example, S‐nitrosylation of HSP90 at Cys589 promotes interaction with glycogen synthase kinase 3β (GSK3β) and increases GSK3β phosphorylation, which contributes to the process of myocardial hypertrophy.^[^ ^38^ ^]^ Genetic or pharmacological inhibition of HSP90 S‐nitrosylation attenuates myocardial fibrosis and adverse ventricular remodeling responses.^[^ ^39^ ^]^ Hyper‐acetylation of HSP90 inhibits the formation of the HSP90‐CX43‐Tom20 complex, thereby mediating mitochondrial damage in cardiomyocytes.^[^ ^40^ ^]^ In addition, HSP90 is methylated by the lysine methyltransferase Smyd2 and stabilizes the scaffolding protein titin to regulate sarcomere assembly.^[^ ^41^ ^]^ Recent studies have shown that inhibition of HSP90 mediated by small molecule inhibitors or gene deletion improves cardiac function in myocardial ischemia,^[^ ^42^ ^]^ chronic heart failure,^[^ ^43^ ^]^ and heart aging.^[^ ^44^ ^]^ However, pharmacological inhibition of HSP90, including 17‐AAG, geldanamycin, 17‐DMAG, and celastrol, is extensive, affects multiple signaling pathways and results in low clinical conversion. Therefore, drug design based on HSP90ab1 Lys72 will be possible for the most effective treatment of post‐ischemic HF.

Under normal conditions, cardiac fibroblasts remain quiescent and maintain ECM homeostasis. After MI, inflammatory cells in the injured area produce large amounts of pro‐inflammatory cytokines and chemokines, and fibroblasts exhibit a pro‐inflammatory phenotype with highly upregulation of Cx3c11, Ccl5, and Csf1, while biosynthetic signaling, including the need for cell membrane synthesis, is reduced.^[^ ^45^ ^]^ This suggests that fibroblasts exhibit anti‐proliferative and anti‐transformation properties during the inflammatory period after myocardial injury. Fibroblasts convert from a pro‐inflammatory state to producing ECM to support the generation of the infarct scar. Excessive ECM accumulation leads to adverse ventricular remodeling. Therefore, well‐healed infarcts require the continued presence of a minimum number of fibroblasts to prevent adverse remodeling that progresses to heart failure.^[^ ^46^ ^]^ Our data suggest that loss of cardiomyocyte SENP1 stimulates fibroblast proliferation and transformation through paracrine effects. However, future studies require complex fibroblast profiling using high‐dimensional platforms to elucidate a deeper understanding of the fibrotic response. The long‐term prognosis after MI depends on the quality and quantity of scar formation. Too little scar leads to inadequate repair with the risk of ventricular rupture, and too much scar can generate a rigid left ventricle. Therefore, intervention of SENP1 on cardiomyocytes at specific times is essential to prevent excessive myocardial fibrosis in patients in the clinic.

So far, six members of the SENPs family with the ability to deSUMOylation have been identified (SENP‐1, −2, −3, −5, −6, −7), SENPs are essential for maintaining the homeostasis of biological responses in the organism by regulating the state of SUMOylation of specific substrates in physiology and pathology. SENP2 is required to regulate embryonic heart development and maintain normal myocardium structure, and studies have confirmed that SENP2 upregulation and increased deSUMOylation in mouse hearts lead to cardiac dysfunction and congenital heart defects by altering cardiomyocyte division.^[^ ^47^ ^]^ And for the treatment of heart failure, targeted reduction of SENP2 has beneficial effects.^[^ ^48^ ^]^ SENP3 and SENP5, which are both nucleolus proteins and specifically bind to SUMO2/3. Previous studies have shown that SENP3 acts as a redox‐sensitive enzyme was highly expressed in vascular smooth muscle cells of remodeled arteries. SENP3 promotes cell proliferation and migration by interacting with β‐catenin and inhibiting its proteasome‐dependent degradation through deSUMOylation of β‐catenin.^[^ ^49^ ^]^ In addition, adenovirus‐mediated overexpression of SENP3 promotes mitochondrial translocation of dynamin‐related protein 1(Drp1) in the myocardium during reperfusion, resulting in impaired intra‐mitochondrial redox status and myocardial damage.^[^ ^50^ ^]^ Similarly, SENP5 mediates mitochondrial fission in the heart by regulating Drp1. Cardiac specific overexpression SENP5 reduces the level of SUMO2/3 conjugated DRP1, which induces increased apoptotic cell death and ultimately leads to cardiomyopathy in adult mice.^[^ ^51^ ^]^ The SENP6 and SENP7 subclasses are both cytoplasmic protein and display a clear proteolytic cleavage preference for SUMO2/3 chain isoforms. Although the role of the polySUMO chain‐cutting enzymes SENP6 and SENP7 in the heart is unclear, recent efforts have been made to investigate specific targets for SENP6 and SENP7. Studies have shown that SENP6 depletion leads to uncoordinated recruitment and persistence of SUMO2/3 at DNA damage sites, resulting in impaired genomic stability.^[^ ^52^ ^]^ Another study^[^ ^53^ ^]^ showed that SENP6 inactivation impairs post‐recombination telomere deSUMOylation, leading to gross chromosome mis‐segregation and mitotic cell death, which suggests a promising application of SENP6 in non‐cardiomyocytes in the heart. Recent studies have shown that SENP7 mediates cellular biochemical processes through metabolic regulation. SENP7 maintains the CD8^+^ T cell metabolic state by mediating deSUMOylation of phosphatase and tensin homolog.^[^ ^54^ ^]^ In addition, Senp7 promotes the lipid titration site of perilipin‐4 (Plin4) by mediating deSUMOylation of Plin4, thereby maintaining lipid metabolism homeostasis in mice.^[^ ^55^ ^]^ Considering that the heart is highly dependent on cellular metabolism to maintain pumping function, targeting SENP7 could be of therapeutic interest to fight heart disease. In this study, we identified SENP1 as a positive regulator of cardiac repair and a potential drug target for the treatment of MI. Moreover, SENPs‐mediated deSUMOylation studies provide a theoretical basis for the pathogenesis and treatment of cardiovascular disease, and targeting to explore the effects and targets of different SENPs subtypes and their potential therapeutic roles may be promising.

In summary, we provide evidence that, SENP1, by promoting HSP90 degradation, prevents ischemia‐mediated myocardial fibrosis. Blocking HSP90ab1 K72‐SUMOylation by a cardiomyocyte‐targeted gene intervention strategy may be an effective novel approach to reverse post‐MI remodeling and attenuate HF progression.

## 4 Experimental Section

A Methods section and expanded Materials are available in the Supporting Information, which involves information on echocardiography measurement, primary mouse cardiomyocyte, fibroblast and endothelial cell isolation and cultivation, neonatal rat ventricular myocyte (NRVMs) isolation, DIA proteome bioinformatics analysis, immunofluorescence, EDU cell proliferation assay, immuno‐histological staining, MASSON's trichrome staining and fibrosis calculation of mouse heart, TUNEL staining, and mass spectrometry, which are listed in the Supporting Information.

### Human Heart Samples

All procedures performed in studies involving human participants were in accordance with the ethical standards of the Ethics Committee of Tianjin chest hospital (IRB‐SOP‐016(F)−001‐02) and with the 1964 Helsinki declaration and its later amendments or comparable ethical standards. Heart samples were obtained from four individuals afflicted with HF and subjected to a trans jugular interventricular septum myocardial biopsy. The cardiac biopsy samples were utilized for pathological evaluation and protein extraction. Table S1 (Supporting Information) provides the detailed information about the patients. The patients/participants provided their written informed consent to participate in this study. The procedures of all human heart tissues were in accordance with the principles of the Helsinki Declaration.

### Animal experiment

All experiments with live animals were approved by the Institutional Animal Care and Use Committee of Tianjin University of Traditional Chinese Medicine, according to the guidelines of the TCM Animal Research Committee (TCM‐LAEC2019105) of Tianjin University of Traditional Chinese Medicine. Mice and rats were kept in a sterile environment with a 12:12 h light/dark cycle (ambient temperature of 23 °C) and fed a standard laboratory diet. Sprague–Dawley (SD) rats (200 ± 20 g) and C57BL6/J mice (20 ± 20 g) were provided by Beijing Vital River Laboratory Animal Technology (Beijing, China). The Myh6Cre mice were purchased from The Jackson Laboratory (IMSR_JAX:0 05657), and the SENP1flox/flox mice were provided by Zhiqiang Liu, Tianjin Medical University^[^ ^56^ ^]^ SENP1flox/floxMyh6Cre were produced by mating SENP1flox/flox mice to Myh6Cre mice. Eight‐week‐old Myh6Cre and SENP1flox/floxMyh6Cre mice were intraperitoneally injected with tamoxifen (30 mg kg^−1^ d^−1^) dissolved in 95% corn oil/5% ethanol for consecutive 5 days. The primers for SENP1flox/flox were: loxP forward, 5′‐AGAGTGAGACCCTGTCTCAACCCAAGC‐3′ and loxP reverse, 5′‐CACACAACTAAGTTAACTGCTGGAAACCAGAGC‐3′, with the expected positions in 300 and 260 bps for SENP1flox/flox mice and wild‐type mice.

### Animal Models of MI

All animals and samples were assigned an alphanumeric designation for blinding purposes. The MI model was induced by permanent ligation of the left anterior descending coronary artery (LAD) as previously described.^[^ ^11^ ^]^ In short, mice were anaesthetized with 1% isoflurane supplemented by a single intraperitoneal injection of 80 mg kg^−1^ ketamine and 7 mg kg^−1^ xylazine. The mice were then intubated with a 16‐gauge intravenous catheter for mechanical ventilation and intubated with a rodent ventilator to maintain a normal steady respiratory rate (≈130 bpm min^−1^). A left‐sided thoracotomy was performed to expose the heart and the LAD was ligated with 6‐0 silk, after which the muscle tissue and skin were sutured and the mice were placed on a heating plate until they recovered from anesthetic. The same procedure was performed without ligation in the sham group.

### In Vivo Gene Therapy

Recombinant adeno‐associated virus (serotype 9) vectors carrying mouse SENP1 (accession number NM_144 851) or HSP90ab1 (accession number NM_0 08302.3 K72R) with a CTNT promoter (AAV9‐CTNT‐SENP1) or (AAV9‐CTNT‐Flag‐HSP90K72R) (Hanbio Inc) to achieve gene overexpression or site mutation in vivo. AAV9‐CTNT‐Vector was used as a negative control. Virus was delivered to mouse heart by tail vein injection (viral titer: 1 × 10^12^ v g mL^−1^, 100 µL per mouse). SENP1 interference was achieved in vivo using a miR30 shRNA‐based lentiviral vector system with a CTNT promoter (AAV9‐CTNT‐shSENP1) (p1: CGTAACGGTAACCAGGATGAA; p2: TTCATCCTGGTTACCGTTACG) (Hanbio Inc). Viral infection efficiency was detected after 2 weeks and MI experiments were performed.

### Cell Model of Ischemic (Hypoxia and Serum Starvation) Injury

For the induction of ischemia (hypoxia and serum starvation)‐mediated injury, adult mouse ventricular cardiomyocytes or neonatal rat cardiomyocytes were isolated and placed in laminin‐coated culture dishes in a normal incubator for 24 h to allow adhesion. Primary cardiomyocytes were then placed in ischemic culture medium (118 mmol L^−1^ NaCl, 24 mmol L^−1^ NaHCO_3_, 20 mmol L^−1^ sodium lactate, 16 mmol L^−1^ KCl, and 10 mmol L^−1^ 2‐deoxyglucose, 2.5 mmol L^−1^ CaCl_2_·2H_2_O, 1.2 mmol L^−1^ MgCl_2_, 1.0 mmol L^−1^ NaH_2_PO_4_, pH adjusted to 6.2),^[^ ^57^ ^]^ and transferred to a 1% O_2_ hypoxic incubator (94% N_2_, 5% CO_2_) for 24 h.

### Single‐Cell Transcriptome Analysis

According to previously published data,^[^ ^11^ ^]^ visualization of individual genes was performed using the FeaturePlot function (Fn and HSP90ab1).

### Sirius Red Staining and Fibrosis Calculation of Mouse Heart

Sirius red staining was performed in accordance with the manufacturer's (Solarbio, Beijing, China). Briefly, heart tissue was fixed in 4% paraformaldehyde solution for 24 h, paraffin embedded and sectioned at 4 µm thickness. The tissue was stained with hematoxylin for 10 min and rinsed under running water for 10 s. Drops of Sirius Red stain were added for 20 min. Photographs were taken using an Olympus BX53 microscope. Fibrotic area of heart was calculated by Image‐Pro Plus 6.0 software (Media Cybernetics, Inc, Rockville, MD, USA).

### RNA Isolation and qRT‐PCR Analysis

Total RNA was extracted from the left ventricle using Trizol reagent. The mRNA levels were detected by quantitative RT–PCR using SYBR Green Master Mix. Relative mRNA levels were determined using the 2^−ΔΔCT^ method, and all data were normalized to gapdh mRNA levels. The PCR primers were obtained from Sangon Biotech. The sequences of the primers used in the current study are listed as follows:

α‐SMA: F: GTCCCAGACATCAGGGAGTAA;
R: TCGGATACTTCAGCGTCAGGA
periostin: F: CGGGAAGAACGAATCATTACA;
R: ACCTTGGAGACCTCTTTTTGC
col1a2: F: AGCCCTGGTTCTCGAGGT
R: CCGGTTGAACCACGATTG
fn: F: ACCGAAGCCGGGAAGAGCAA
R: GGTCCGTTCCCACTGCTGATTTATC
hsp90ab1: F: GTCCGCCGTGTGTTCATCAT
R: GCACTTCTTGACGATGTTCTTGC
gapdh: F: GGCACAGTCAAGGCTGAGAATG
R: ATGGTGGTGAAGACGCCAGTA
rat senp1: F: CTCAGGCTTTCCAGAGGACC
R: ACTGCTTGTAGAACCCGTGA
rat gapdh: F: ATCACCATCTTCCAGGAGCGA
R: AGCCTTCTCCATGGTGGTGAA

### Chromatin Immunoprecipitation (CHIP) Assay

Primary cardiomyocytes were cross‐linked with 1.1% formaldehyde at 37 °C for 10 min, burst with glycine, and then fragmented using a ultrasonic cell disruptor to produce 200–600 bp DNA fragments. Immunoprecipitation was performed using anti‐STAT3 antibody (Cell Signaling Technology, Boston, USA) and normal rabbit IgG (Cell Signaling Technology, Boston, USA) as controls. Precipitated DNA was detected with specific primers to detect STAT3 binding to the Fn promoter (forward:5′‐ CTCAAGATGCTCAGGGGTCC‐3′; reverse:5ʹ‐ ATTTGCTGAGCCTGCCTCTT‐3ʹ).

### Plasmid Transfection

Human Embryonic Kidney 293T (HEK293T) cells were purchased from Procell (Procell, Wuhan, China) and cultured in Dulbecco's Modified Eagle Medium with 10% fetal bovine serum (FBS) and 1‰ streptomycin at 37 °C in the 5% CO_2_–95% air atmosphere. Plasmid transfection of HEK293t was performed using Lipofectamine 2000 (ThermoFisher Scientific, MA, USA) according to manufacturer's protocol. Plasmid was used to transfect HEK293T at ≈60–70% confluence.

### Immunoprecipitation and Western Blotting

Immunoprecipitation was performed in accordance with the manufacturer's protocol (#88805, Thermo Fisher Scientific). Briefly, fresh heart tissues or cells were lysed. Immunoprecipitation was performed in accordance with the manufacturer's protocol (Thermo Fisher Scientific, MA, USA). Tissue or cells were lysed on ice for 30 min, followed by vortexing at 14 000 rpm at 4 °C for 15 min. Beads were incubated with specific antibodies at room temperature for 1 h on a vortex (Table S6, Supporting Information). The lysates were then incubated with the antibody bead‐antibody complexes for 2 h. The resulting protein complexes were separated and subjected to Western blot experiments. Briefly, protein homogenates were separated by SDS‐PAGE, transferred to PVDF membranes, blocked with 5% skimmed milk for 1 h and incubated with specific antibodies overnight at 4 °C. The membranes were then incubated with secondary antibodies for 1 h and visualized using enhanced chemiluminescence reagent (Thermo Fisher Scientific, MA, USA).

### Statistical Analysis

Results are expressed as the mean ± SEM. All statistical analyses were performed with GraphPad Prism 7.0 software. The Shapiro–Wilk test was used to test the normality of all data obtained from the in vivo study. For comparisons between two groups, analyses were performed using the unpaired Student *t*‐test (for normally distributed data) or the Mann–Whitney test (for not normally distributed data). Comparisons between multiple groups were performed using one‐way analysis of variance (ANOVA) followed by Tukey's post hoc multiple comparison test or two‐way ANOVA followed by Sidak's post hoc multiple comparison test. When two conditions were considered between groups, two‐way ANOVA with appropriate post‐hoc correction was used. All experiments were performed independently, *p *< 0.05 was considered statistically significant.

### Ethics Approval Statement

All experiments with live animals were approved by the Institutional Animal Care and Use Committee of Tianjin University of Traditional Chinese Medicine, according to the guidelines of the TCM Animal Research Committee (TCM‐LAEC2019105) of Tianjin University of Traditional Chinese Medicine.

## Conflict of Interest

The authors declare no conflict of interest.

## Author Contributions

Z.L. and X.B. contributed equally to this work. Z.H.L., X.Y.B., G.W.F., X.Z.L., and X.H.F. conceived the project. Z.H.L., X.Y.B., L.L., C.F., Y.W., and J.Y.N. contributed to cell extraction, cardiac cell coculture, confocal microscopy, RNA and protein extraction, and gels. S.L, D.D.L., and Y.X.L. responsible for the breeding and genotyping of mice. C.R.M., T.Y., and X.L.X. generated and analyzed the proteomics data. L.L., N.X., and Y.X.W. collected and analyzed the single‐cell sequencing data. C.Y.Z. and X.F.M. collected and analyzed all mouse echocardiography data. L.L., C.F., X.Z.L., and X.M.G. provided key reagents and identified the patient information used. Z.H.L. and X.Y.B. compiled the final figures with contributions from the other authors. Z.H.L., G.W.F., and X.H.F. wrote the manuscript. All authors approved the final version of the manuscript.

## Supporting information

Supporting Information

## Data Availability

The data that support the findings of this study are available in the supplementary material of this article.
