# Supplementary material for: SENP1‐Mediated HSP90ab1 DeSUMOylation in Cardiomyocytes Prevents Myocardial Fibrosis by Paracrine Signaling
Source: Adv Sci (Weinh). 2024 Jul 11;11(34):2400741. doi: 10.1002/advs.202400741 (PMC11425837; doi:10.1002/advs.202400741)
Supplement: Supplementary file 1 — Supporting Information [file ADVS-11-2400741-s001.docx]

***Supplementary Materials for***

**Title:** **SENP1-mediated HSP90ab1 DeSUMOylation in Cardiomyocytes Prevents Myocardial Fibrosis by Paracrine Signaling**

**Authors:** Zhihao Liu^1,5#^, Xiyun Bian^2,8#^, Lan Li^5^, Li Liu^1,5^, Chao Feng^7^, Ying Wang^2,8^, Jingyu Ni^1^, Sheng Li^5^, Dading Lu^2,8^, Yanxia Li^2,8^, Chuanrui Ma^1^, Tian Yu^2,8^, Xiaolin Xiao^2,8^, Na Xue^2,8^, Yuxiang Wang^2,8^, Chunyan Zhang^2,8^, Xiaofang Ma^2,8^, Xiumei Gao^5,6^, Xiaohui Fan^3,4*^, Xiaozhi Liu^2,8*^, Guanwei Fan^1,5,6*^

**Affiliations:**

^1^ First Teaching Hospital of Tianjin University of Traditional Chinese Medicine, National Clinical Research Center for Chinese Medicine Acupuncture and Moxibustion, Tianjin 300193, China.

^2^ Tianjin Key Laboratory of Epigenetics for Organ Development in Preterm Infants, the Fifth Central Hospital of Tianjin, Tianjin 300450, China.

^3^ Pharmaceutical Informatics Institute, College of Pharmaceutical Sciences, Zhejiang University, Hangzhou, Zhejiang, 310058, China.

^4^ National Key Laboratory of Chinese Medicine Modernization, Innovation Center of Yangtze River Delta, Zhejiang University, Jiaxing 314100, China.

^5^ State Key Laboratory of Component-Based Chinese Medicine, Tianjin 301617, China

^6^ Haihe Laboratory of Modern Chinese Medicine, Tianjin 301617, China.

^7^ Department of Cardiology, Tianjin Chest Hospital, Tianjin 300051, China.

^8^ Central Laboratory, Tianjin Fifth Central Hospital, Tianjin 300450, China.

*Corresponding authors: Pro. X. Fan(fanxh@zju.edu.cn); Pro. X. Liu(lxz7997@126.com); Pro. G. Fan(guanwei.fan@tjutcm.edu.cn).

#Authors contributed equally to the work.

**Supplemental Methods**

***Echocardiography measurement***

To assess cardiac function in mice, the Vevo2100 ultrasound imaging system (Visualsonics, US) was used to measure left ventricular function in mice. Briefly, the chest hair was removed and ultrasound gel was applied to the chest surface to obtain optimal cardiac visibility. The mice were held without anesthesia during the ultrasound procedure. B-mode and M-mode images were viewed in the short-axis view. Left ventricular ejection fraction (EF), shortening fraction (FS), left ventricular systolic internal diameters (LVIDs), and left ventricular diastolic internal diameters (LVIDd) were measured.

***Primary mouse cardiomyocyte, fibroblast and endothelial cell isolation and cultivation***

Adult cardiomyocytes were isolated from the ventricles of wild-type (C57BL/6J), Myh6^Cre^, SENP1 deletion, and SENP1 overexpression mice using previously described methods with some modifications. Briefly, mice were anesthetized and the thoracic cavity is opened to expose the heart. After dissection of the descending aorta, the heart was flushed by injecting 8 ml of EDTA buffer into the right ventricle. The heart was quickly transferred to a Petri dish containing fresh EDTA solution. The heart was perfused sequentially via the aorta with EDTA buffer, perfusion buffer and collagenase buffer to digest the heart tissue. The ventricles were separated and gently torn into irregular pieces of approximately 1 mm using forceps. The tissue fragments were gently aspirated with a 5 ml syringe, followed by the addition of 5 ml of termination buffer containing serum. The cell suspension was passed through a 100 μm filter membrane and the cells were subjected to 4 rounds of gravitational sedimentation with a calcium-containing gradient solution to bring the calcium concentration to physiological levels. Cardiomyocytes were cultured in dishes precoated with laminin (5μg/mL). After 1 h and every 48 h thereafter, the media were replaced with fresh, prewarmed culture media. For fibroblasts, mice were anesthetized and the thorax opened to remove the heart. The hearts were removed under aseptic conditions and rinsed in D-Hank's solution. Five times the volume of digestion solution was added to the excised hearts, which were blown repeatedly in a 37°C water bath for 3-5 min, allowed to stand and the supernatant discarded after natural sedimentation. The remaining precipitate was then added to a fivefold volume mixture of 0.08% trypsin and 0.1% collagenase II, pulsed repeatedly for approximately 10 minutes, centrifuged and the precipitate resuspended in DMEM containing 20% BSA. After 60-90 minutes, the culture medium was changed and the walled fibroblasts were cultured.

For endothelial cells, hearts from three mouse were removed aseptically in each group, rinsed in DMEM supplemented with 20% FBS, minced finely with scissors for 1min, and digested in 25 mL of type I collagenase (2 mg/mL) at 37°C for 45 minutes with occasional agitation. The cellular digest was filtered through sterile 70-µm disposable cell strainer, centrifuged at 400g for 10 minutes, and washed twice with cold PBS containing 0.1% BSA; the cell pellet was resuspended in 2mL of cold PBS containing 0.1% BSA. Dynabeads M-450 sheep anti-rat IgG pre-incubated overnight with rat anti-mouse PECAM-1 antibody were added into the cell suspension at 15µl of beads per 1 ml of cell suspension. After 10 minutes at room temperature with occasional agitation, the bead-bound cells were separated with a magnetic separator, washed five times with DMEM supplemented with 20% FBS, and resuspended in DMEM supplemented with 20% FBS, 100 µg/ml Heparin, 10% endothelial cell growth medium (ECGM), and 25 mol/L HEPES, and then performed Western blotting.

***Neonatal rat ventricular myocyte (NRVMs) isolation***

Primary neonatal rat ventricular cardiomyocytes (NRVMs) were isolated from the ventricles of 1-day-old SD rats. Briefly, the neonatal rat ventricles were minced and digested with 3ml collagenase and 1.5ml pancreatic enzyme (0.05%) in 37℃ water bath for 8min. The supernatant was discarded after natural sedimentation. Add 3 ml collagenase and 1.5 ml trypsin and digest at 37°C for 10 minutes, remove the supernatant and centrifuge at 3000 rpm for 5 minutes. Transfer the cell sediment to a medium containing 10% fetal bovine serum in DMEM medium, add 3 ml collagenase and 1.5 ml trypsin to the remaining sediment, blow and digest at 37°C for 10 minutes. Repeat the above steps 4-5 times until digestion of the tissue block is complete. After 2-3 hours in the incubator, the fibroblasts are allowed to adhere to the wall and the medium is gently blown, all the supernatant from the medium is transferred to a centrifuge tube and centrifuged for 5 minutes (3000 rpm), the supernatant is discarded, DMEM medium containing 10% calf serum and Brdu (10mM) was used for cell culture.

***DIA proteome bioinformatics analysis***

For DIA proteome bioinformatics analysis, all sample peptide mixtures were redissolved in buffer A (buffer A: 20 mM ammonium formate in water, adjusted to pH 10.0 with ammonia) and separated at high pH using an Ultimate 3000 system (Thermo Fisher scientific) connected to a reversed-phase column (XBridge C18 column, 4.6 mm x 250 mm, 5 μm) for high pH separation using a linear gradient from 5% B to 45% B in 40 min (B: 80% ACN with 20 mM ammonium formate adjusted to pH 10.0 with ammonia). The column was equilibrated under initial conditions for 15 min, with the column flow rate maintained at 1 mL/min and the column temperature at 30°C.

The desalted lyophilised peptides were re-dissolved in solvent A (A: 0.1% aqueous formic acid solution) and analysed by LC-MS/MS using an on-line nano-HPI source. The complete system consisted of an Orbitrap Lumos mass spectrometer (Thermo Fisher Scientific) in tandem with an EASY-nLC 1200 system. A total of 3μl was sampled (analytical column Acclaim PepMap C18, 75 μm x 25 cm) in a 120 min gradient separation: 5% B to 35% B (B: 0.1% formic acid ACN solution). The column flow rate was controlled at 200 nL/min and the electrospray voltage was 2 kV. The Orbitrap Lumos mass spectrometer was operated in data dependent acquisition mode, automatically switching between MS and MS/MS acquisition. The mass spectrometry parameters were set as follows: (1) MS: Scan range (m/z): 350-1500; Resolution: 120,000; AGC target: 4e5; Max injection time: 50 ms; Dynamic exclusion time: 30 s; (2) HCD-MS/MS: Resolution: 15,000; AGC target= 5e4; Max injection time: 35ms; collision energy: 32. DIA data acquisition was then performed, each sample was suspended by adding 30μL of solvent A (A: 0.1% aqueous formic acid solution), and analysed by on-line electrospray tandem mass spectrometry. The complete experimental system consisted of an Orbitrap Lumos mass spectrometer (Thermo Fisher Scientific,) connected to an EASY-nLC 1200 system. A total of 3μL was sampled (analytical column Acclaim PepMap C18, 75μm x 25 cm) in a 120 min gradient separation: 5% B to 35% B (B: 0.1% formic acid ACN solution). The column flow rate was controlled at 200nL/min and the electrospray voltage was 2 kV. Mass spectrometry parameters were set as follows: (1) MS: Scan range (m/z): 350-1500; Resolution: 120,000; AGC target: 4e6; maximum injection time: 50ms; (2) HCD-MS/MS: resolution: 30. 000; AGC target: 1e6; collision energy: 32; energy increase: 5%. (3) Variable window acquisition with 60 windows set up and overlapping serial ports set up with 1 m/z overlap per window.

***Immunofluorescence***

For histological immunofluorescence staining, heart tissue was fixed in 4% paraformaldehyde solution for 24 h, paraffin embedded and sectioned at 4μm thickness. 10 mM sodium citrate (pH 6.0) was used for antigen repair. 0.1% Triton X-100 was infiltrated for 10 minutes, washed 3 times with PBST and then blocked with 3% bovine serum albumin for 1 hour. Immunostaining of CTNT, SENP1, α-Actinin, collagen type I, α-SMA, FN and p-STAT3 using anti-CTNT (# ab91695, Abcam, 1:300), anti-SENP1 (# ab108981, Abcam, 1:300), anti-α-Actinin (# 23660-1-AP, Proteintech, 1:200), anti-collagen type I (# 14695-1-AP, Proteintech, 1:200), anti-α-SMA (# ab7817, Abcam, 1:200), anti- FN (# 15613-1-AP, Proteintech, 1:200), and anti-p-STAT3 (# ab76315, Abcam, 1:300). Primary antibodies were performed on tissue sections overnight at 4 °C. Then the sections were stained with goat anti-rabbit IgG H&L (DyLight® 488) (# ab150077, Abcam, 1:500), goat anti-mouse IgG H&L (DyLight® 594) (# ab150116, Abcam, 1:500), or goat anti-mouse IgG H&L (DyLight® 488) (# ab150117, Abcam, 1:500), goat anti-rabbit IgG H&L (DyLight® 594) (#ab150084, Abcam, 1:500) for 40 minutes at 37℃, and 4',6-diamidino-2-phenylindole (#ab104139, Abcam) was used for nuclear localization. The stained slides were photographed with a confocal laser scanning microscope (LSM 800, ZEISS).

For cell immunofluorescence staining, cell was fixed in 4% paraformaldehyde solution for 40 minutes, 0.1% Triton X-100 was infiltrated for 10 minutes, washed 3 times with PBST and then blocked with 3% bovine serum albumin for 1 hour. Immunostaining of α-SMA and S100a4 using anti-α-SMA (# ab7817, Abcam, 1:200), anti-S100a4 (# ab124805, Abcam, 1:300). Primary antibodies were performed on tissue sections overnight at 4 °C. Then the sections were stained with goat anti-rabbit IgG H&L (DyLight® 488) (# ab150077, Abcam, 1:500), goat anti-mouse IgG H&L (DyLight® 594) (# ab150116, Abcam, 1:500) for 40 minutes at 37℃, and 4',6-diamidino-2-phenylindole (#ab104139, Abcam) was used for nuclear localization. The stained slides were photographed with a confocal laser scanning microscope (LSM 800, ZEISS). For the determination of positively stained cells, images were analyzed using Image-Pro Plus 6.0 software (Media Cybernetics, Inc, Rockville, MD, USA).

***EDU cell proliferation assay***

EDU cell proliferation assay was performed in accordance with the manufacturer’s (# C0078S, Beyotime). Briefly, At the end of the experimental cycle of cells cultured in 6-well plates, EDU working solution (10μM) was prepared and the cells were incubated for 2 hours in a cell incubator. The culture medium was removed and 1 ml of 4% paraformaldehyde was added and fixed for 15 minutes at room temperature. Cells were incubated with 0.3% Triton X-100 for 10-15 minutes at room temperature. 1 ml washing solution was added and the cells were washed 2 times. EDU reaction solution was then added and the cells were incubated for 30 minutes at room temperature and protected from light. And 4',6-diamidino-2-phenylindole (#ab104139, Abcam) was used for nuclear localization. The stained slides were photographed with a confocal laser scanning microscope (LSM 800, ZEISS). For the determination of positively stained cells, images were analyzed using Image-Pro Plus 6.0 software (Media Cybernetics, Inc, Rockville, MD, USA).

***Immuno-histological staining***

For immuno-histological staining, heart tissue was fixed in 4% paraformaldehyde solution for 24 h, paraffin embedded and sectioned at 4μm thickness. 10 mM sodium citrate (pH 6.0) was used for antigen repair. 0.1% Triton X-100 was infiltrated for 10 minutes, 3%H2O2 was incubated at room temperature for 20 min and then blocked with 3% bovine serum albumin for 1 hour. Immunostaining of collagen type I using anti-collagen type I (# 14695-1-AP, Proteintech, 1:200). Primary antibodies were performed on tissue sections overnight at 4 °C. Add 100μL goat anti-mouse/rabbit IgG HRP (ZSGB-BIO, PV-6000, Beijing, China) polymer and incubate at 37 ° C for 30 minutes. Then add fresh DAB color solution and incubate at room temperature for 5 minutes. Pictures were taken with an Olympus BX53 microscope. Collagen positively stained area of heart was calculated by Image-Pro Plus 6.0 software (Media Cybernetics, Inc, Rockville, MD, USA).

***MASSON’s trichrome staining and Fibrosis calculation of mouse heart***

MASSON’s trichrome staining was performed in accordance with the manufacturer’s (# G1340, Solarbio, Beijing, China). Briefly, heart tissue was fixed in 4% paraformaldehyde solution for 24 h, paraffin embedded and sectioned at 4μm thickness. Weigert solution A was mixed with Weigert solution B and stained for 10 minutes. Acidic ethanol and Masson bluing solution was added. Ponceau S staining solution was added for 10 minutes and aniline blue for 2 minutes. Photographs were taken using an Olympus BX53 microscope. Fibrotic area of heart was calculated by Image-Pro Plus 6.0 software (Media Cybernetics, Inc, Rockville, MD, USA).

***TUNEL staining***

TUNEL staining was performed in accordance with the manufacturer’s (# C1086, Beyotime, Beijing, China). Briefly, heart tissue was fixed in 4% paraformaldehyde solution for 24 h, paraffin embedded and sectioned at 4μm thickness. Add 20µg/ml Protease K (# ST532, Beyotime, Beijing, China) without DNase. Add 50ul of TUNEL test solution to each sample and incubate at 37℃ for 60 min. And 4',6-diamidino-2-phenylindole (#ab104139, Abcam) was used for nuclear localization. The stained slides were photographed with a confocal laser scanning microscope (LSM 800, ZEISS). The excitation wavelength range is 450-500nm and the emission wavelength range is 515-565nm (green fluorescence). For the determination of positively stained cells, images were analyzed using Image-Pro Plus 6.0 software (Media Cybernetics, Inc, Rockville, MD, USA).

***Mass spectrometry***

SUMO1 or SENP1 magnetic beads were used to purify proteins from primary cardiomyocytes. This was followed by SDS-PAGE gel electrophoresis, and coomassie brilliant blue staining. Liquid chromatography with tandem mass spectrometry analysis was performed on a Q Exactive mass spectrometer (Thermo Scientific) that was coupled to Easy nLC (Thermo Fisher Scientific) for 60 minutes. After reduction and alkylation, the samples were added to trypsin (1:50 mass ratio) and digested at 37°C for 20 hours. Mass spectrometry was then performed (A solution was 0.1% formic acid in water, B solution was 0.1% formic acid in acetonitrile (84% acetonitrile). After the column was balanced with 95% of the A solution, the sample was loaded onto the trap column by the autosampler. Mass-to-charge ratios of peptides and peptide fragments were collected as follows: 20 fragment profiles (MS2 scan) were collected after each full scan. The Proteome Discoverer 1.4 software was used to search the databases and the final results were obtained for the identified proteins.

**Supplementary Figures**

**
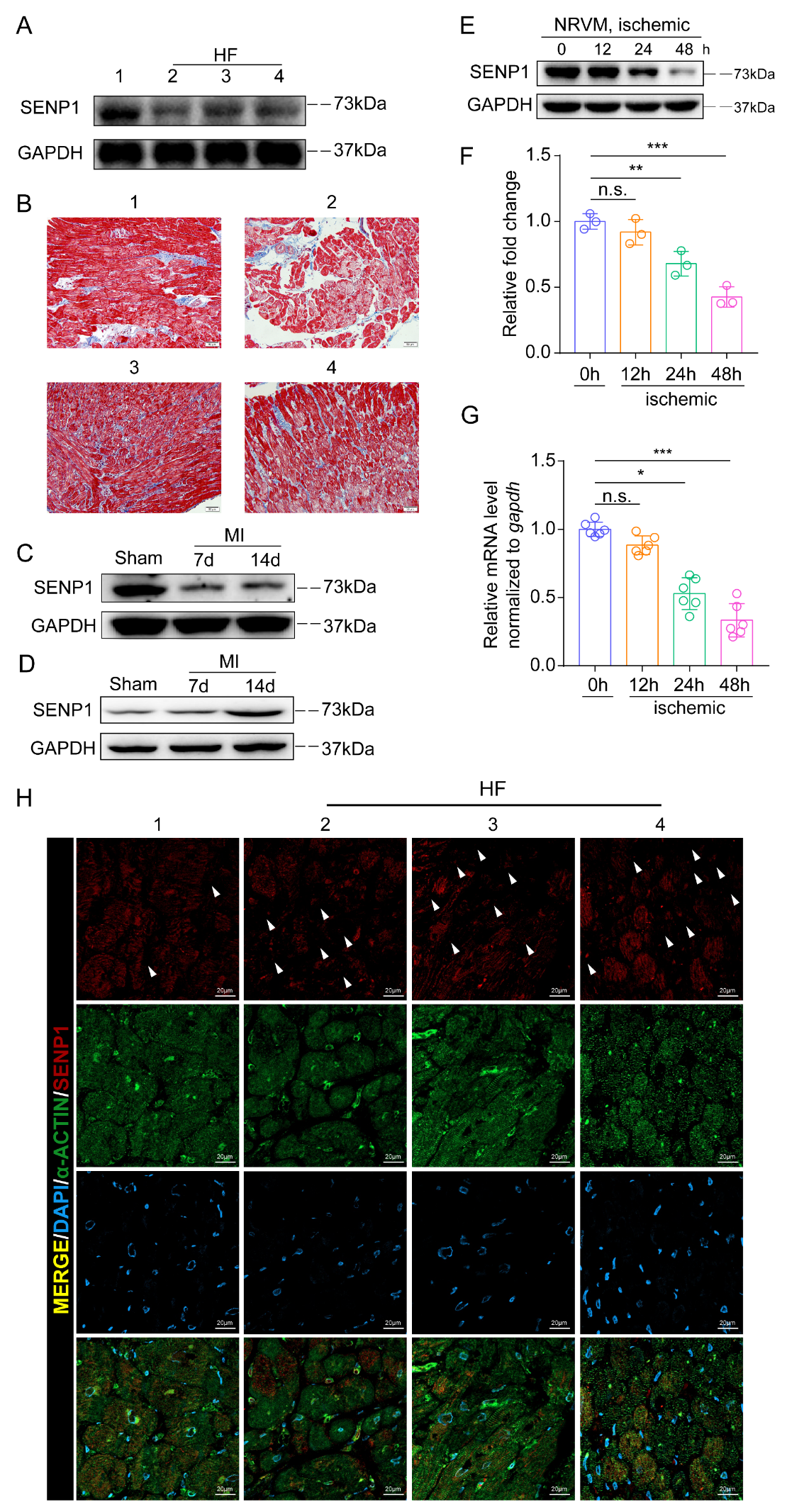
**

**Figure S1.** **Expression of SENP1 (Sentrin-specific protease 1) after myocardial injury. (A)** Immunoblotting of SENP1 protein levels in human heart tissue samples; **(B)** Representative images of heart tissue stained with Masson's trichrome staining showing degrees of fibrosis in human samples; **(C)** Western blotting conducted on cardiomyocytes; **(D)** Western blotting conducted on endothelial cell, isolated from mice heart; **(E)** Representative immunoblotting of SENP1 protein levels in neonatal rat ventricular myocytes (NRVMs) under normal and ischemic (hypoxia and serum starvation) conditions from 12 to 48 hours; **(F)** Quantitative analyses of SENP1 protein; n=3 in each group; **(G)** SENP1 mRNA expression in NRVMs under normal and ischemic conditions from 12 to 48 hours; n=6 in each group. **(H)** Immunofluorescent staining of SENP1 (red) and α-ACTIN (cardiomyocytes marker, green) was performed in human heart tissue samples. Data are shown as mean ± SEM. *p<0.05, **p<0.01, and ***p<0.001, using one-way ANOVA followed by Tukey post hoc multiple comparisons test **(F),** one-way ANOVA followed by the Dunn post hoc multiple comparisons test **(G).**

**Figure S2**

**
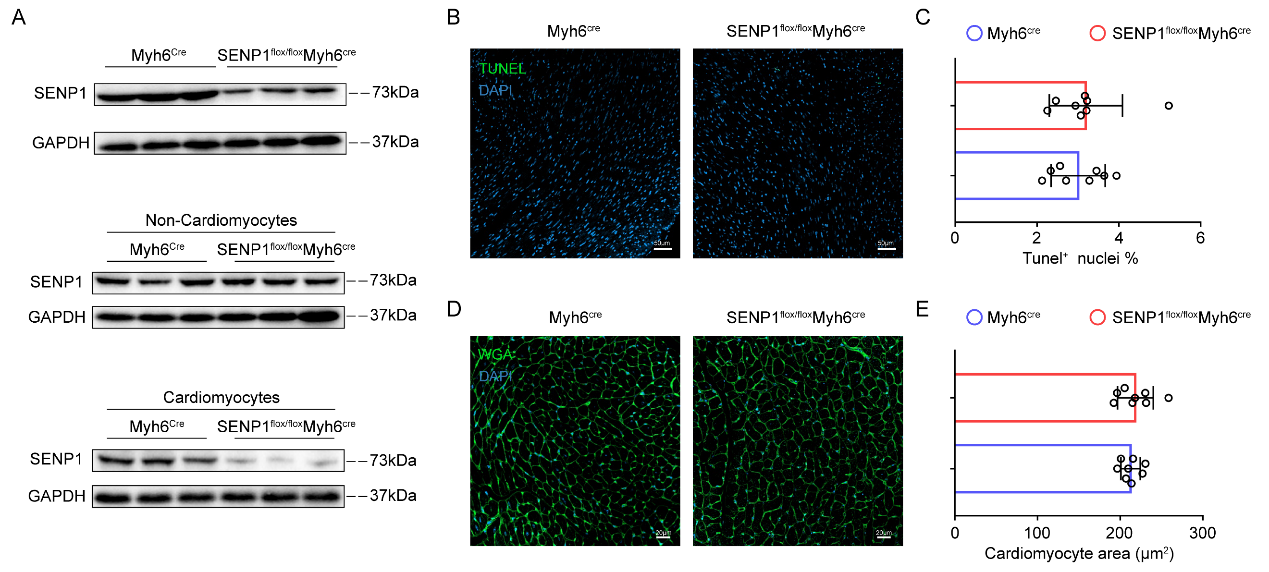
**

**Figure S2. Generation of cardiomyocyte-specific SNEP1 (Sentrin-specific protease 1) knockout mice.** (A) Immunoblots for SENP1 in primary cardiomyocytes and non-cardiomyocytes from SENP1^flox/flox^Myh6^cre^ mice and Myh6^cre^ mice; **(B)** Tunel staining for cardiac sections of SENP1^flox/flox^Myh6^cre^ mice and Myh6^cre^ mice; scale bar, 50 μm; **(C)** Quantitative analyses of Tunel positive areas in DAPI positive cells per field; n=8 in each group; **(D)** Wheat germ agglutinin (WGA) staining for cardiac sections of SENP1^flox/flox^Myh6^cre^ mice and Myh6cre mice; scale bar, 20 μm; **(E)** Quantitative analyses of average area of individual cardiomyocytes per field; n=8 in each group. Data are shown as mean ± SEM. Using Mann-Whitney U test **(C)**, unpaired t test **(E)**.

**Figure S3**

**
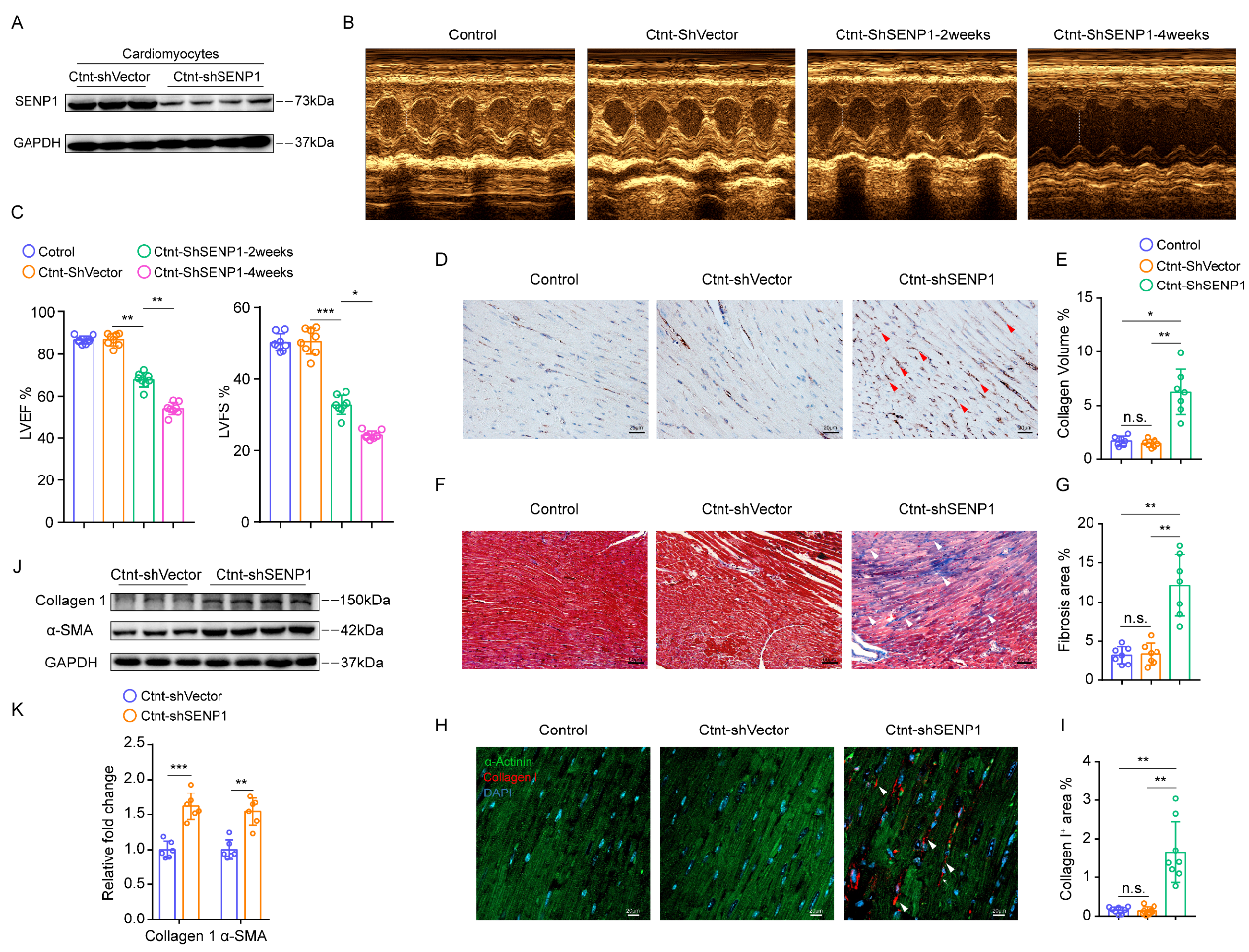
**

**Figure S3. Adeno-associated virus (AAV)-mediated cardiomyocyte-specific SENP1 (Sentrin-specific protease 1) deficiency induces progressive cardiac dysfunction and myocardial fibrosis. (A)** Immunoblots for SENP1 in primary cardiomyocytes from CTNT (cardiac troponin T)-shVector and CTNT-shSENP1 mice; **(B)** Representative echocardiographic images of CTNT-shSENP1 mice; **(C)** Left ventricular ejection fraction (LVEF) and left ventricular fraction shortening (LVFS) measured by 2-dimensional echocardiography on mice, at different time points (baseline, 2 and 4 weeks after injection); n=8 in each group; **(D)** Representative images of left ventricle stained with Collagen I staining showing degrees of fibrosis in mice; scale bar, 20 μm; **(E)** Quantification of collagen I positive areas per field; n=7 in each group; **(F)** Representative images of left ventricle stained with Masson's trichrome staining showing degrees of fibrosis in mice; scale bar, 50 μm; **(G)** Quantitative summary of changes in fibrosis in the hearts of CTNT-shSENP1 mice compared with the CTNT-shVector mice; n=7 in each group; **(H)** Immunofluorescence staining for collagen I–positive (red) and α-actin-positive (green) cardiac sections; scale bar, 20 μm; **(I)** Quantification of collagen I positive areas per field; n=8 in each group; (**J-K**) Immunoblots for ECM (extracellular matrix) proteins, including collagen 1, and α-SMA, in CTNT-shSENP1 and CTNT-shControl mice heart tissues **(J),** and quantitative analyses are plotted in **(K)**. n=6 in each group. Data are shown as mean ± SEM. *p<0.05, **p<0.01, and ***p<0.001, using one-way ANOVA followed by Tukey post hoc multiple comparisons test**(C)**, one-way ANOVA with Kruskal-Wallis test **(E,G**, **I)**, unpaired t test was utilized in **(K)**.

**Figure S4**

**
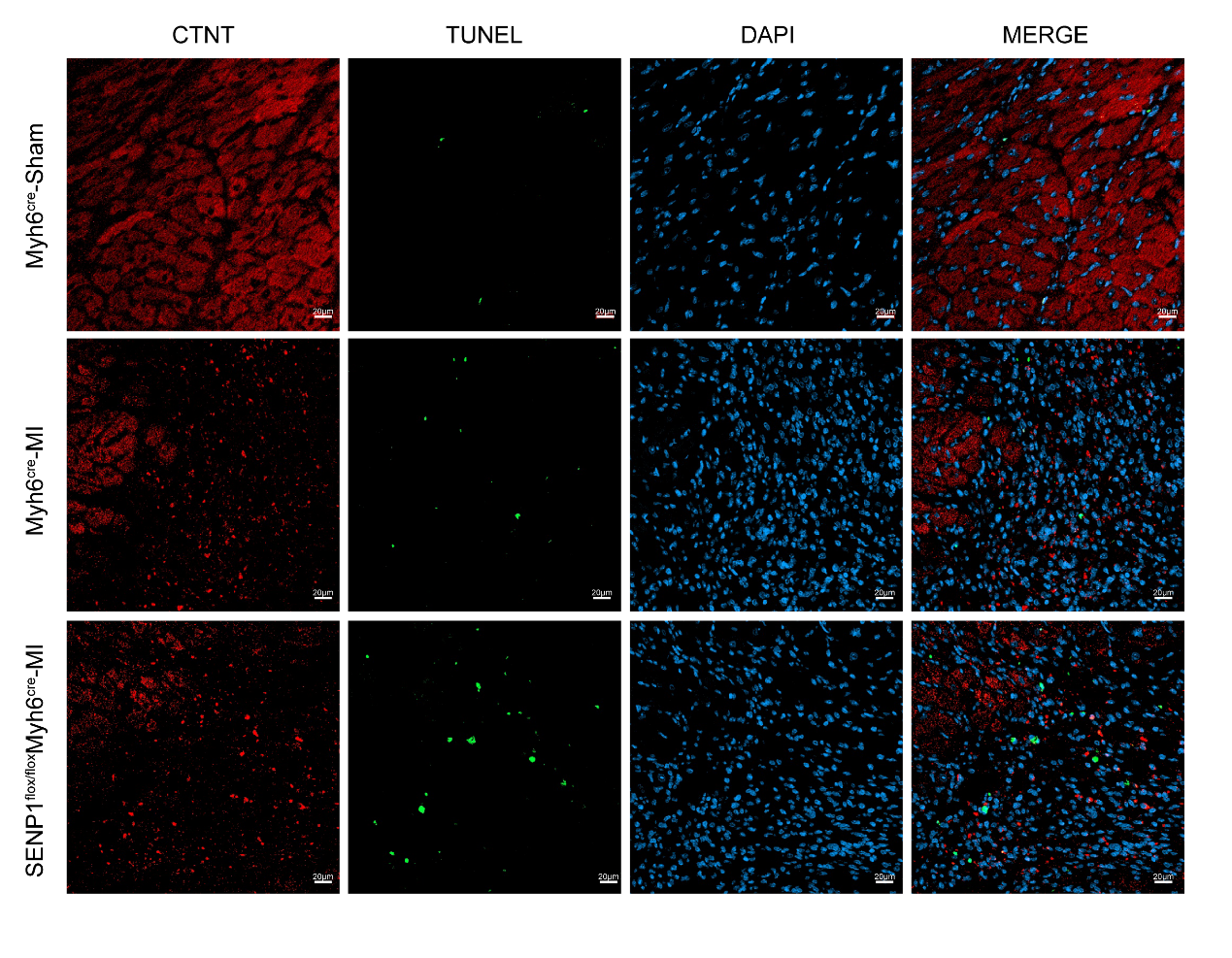
**

**Figure S4. Loss of SENP1 (Sentrin-specific protease 1) promotes cardiomyocyte apoptosis.** Tunel staining for cardiac sections of SENP1^flox/flox^Myh6^cre^ mice and Myh6^cre^ mice 7 days after myocardial infarction (MI); scale bar, 50 μm.

**Figure S5**

**
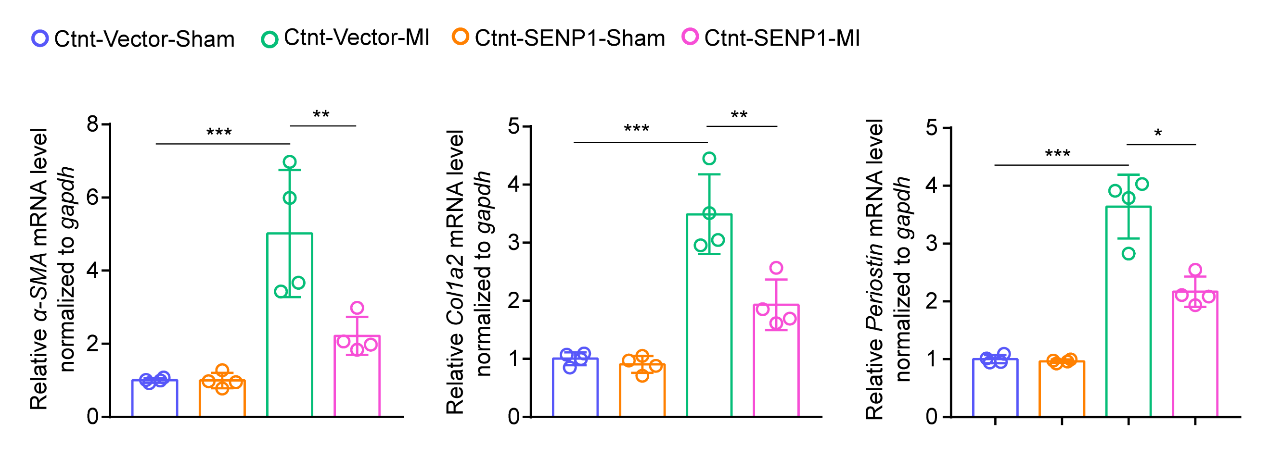
**

**Figure S5.** **Cardiomyocyte specific SENP1 (Sentrin-specific protease 1) overexpression reduces myocardial fibrosis after MI (myocardial infarction).** *α-SMA*, *Col1a2*, and *periostin* mRNA expression in mouse heart tissues; n=4 in each group. Data are shown as mean ± SEM. *p<0.05, **p<0.01, and ***p<0.001, using one-way ANOVA followed by Tukey post hoc multiple comparisons test.

**Figure S6**

**
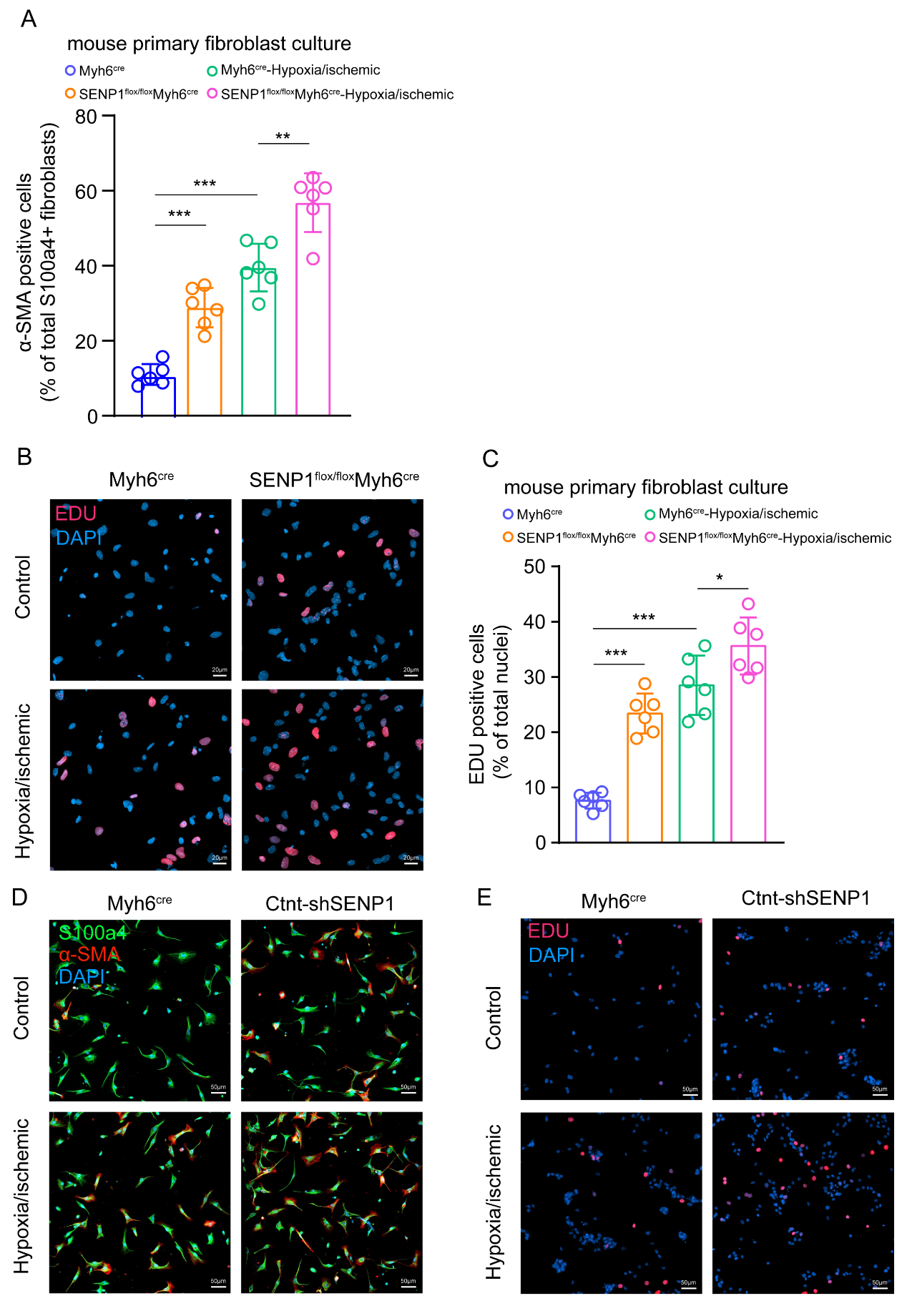
**

**Figure S6****. Conditioned medium collected from Adeno-associated virus (AAV)-mediated SENP1 (Sentrin-specific protease 1) deficiency in cardiomyocytes increases the differentiation and proliferation of fibroblasts.** **(A)** Quantification of S100a4-α-SMA double positive cells per field (n=6); **(B)** Immunofluorescence staining for EDU^+^ (red) primary fibroblasts **(B)** (scale bar: 20 μm) and quantification of EDU^+^ cells per field **(C)** (n=6); **(D)** Immunofluorescence staining for S100a4 (S100 Calcium Binding Protein A4)–positive (green) and α-SMA–positive (red) NIH-3T3 fibroblasts; scale bar, 50 μm; **(E)** Immunofluorescence staining for EDU–positive (red) NIH-3T3 fibroblasts; scale bar, 50 μm. Data are shown as mean ± SEM. **p*<0.05, ***p*<0.01, and ****p*<0.001, using two-way ANOVA followed by Sidak post hoc multiple comparisons test **(A, C).**

**Figure S7**

**
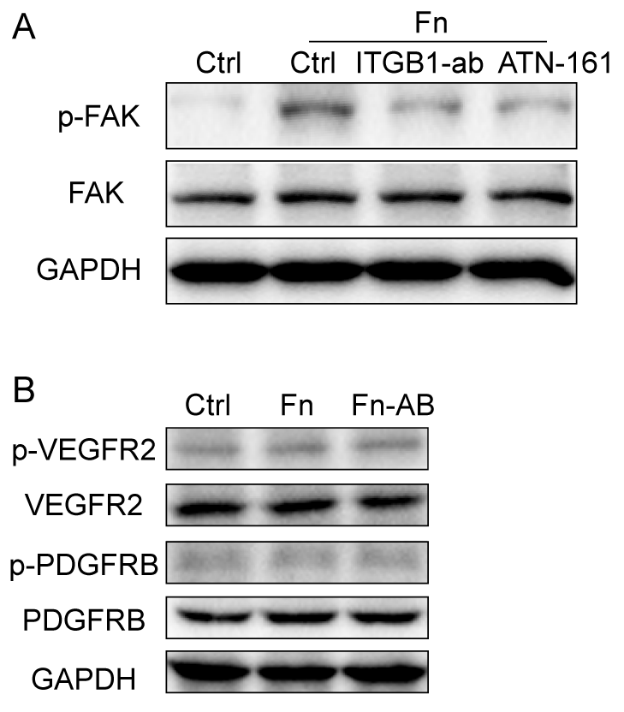
**

**Figure S7. Fn activated FAK through integrin signaling rather than VEGFR and PDGFRB signaling.** **(A)** Immunoblots for p-FAK and FAK in fibroblasts; **(B)** Immunoblots for p-VEGFR2/VEGFR2 and p-PDGFRB/PDGFRB in fibroblasts.

**Figure S8**

**
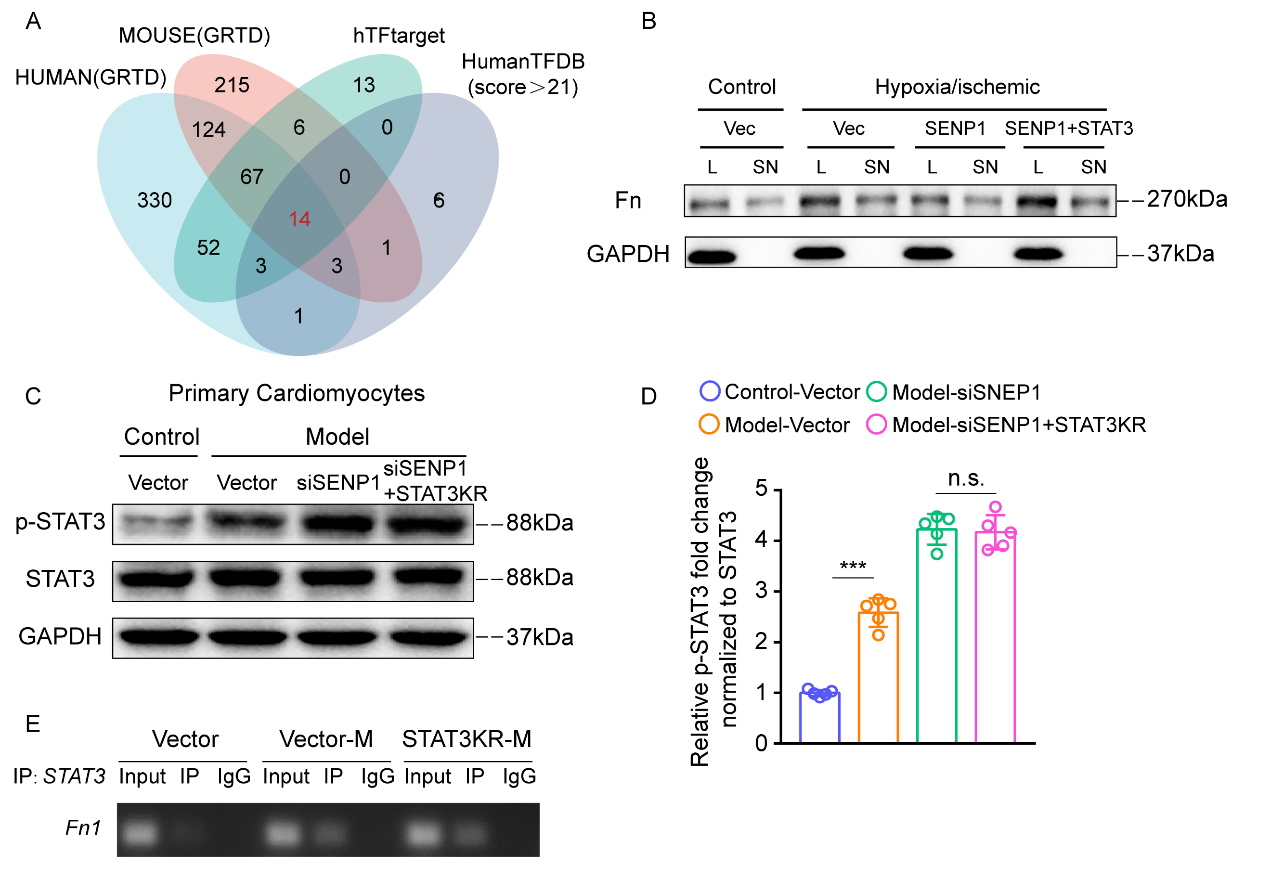
**

**Figure S8. Transcriptional regulation of Fn by SENP1 is mediated by STAT3. (A)** The 14 transcription factors (TFs) bound to the Fn promoter were identified using the GTRD database, the hTFtarget database, and the HumanTFDB database (binding score >21); (**B**) Immunoblots for Fn in mouse primary cardiomyocytes and conditioned medium from adenovirus-transfected mice; **(C-D)** Immunoblots for STAT3 and p-STAT3 in primary cardiomyocytes (normal and ischemic) after transfected with the indicated plasmids (Lys451 site mutation of STAT3) **(C).** Quantitative analyses are plotted in **D**. n=5 in each group; **(E)** The DNA fragment derived from mouse primary cardiomyocytes corresponding to the Fn promoter enriched by STAT3 binding was evaluated by agarose gel electrophoresis. Data are shown as mean ± SEM. ***p<0.001, using one-way ANOVA followed by the Dunn post hoc multiple comparisons test **(D).**

**Figure S9**

**
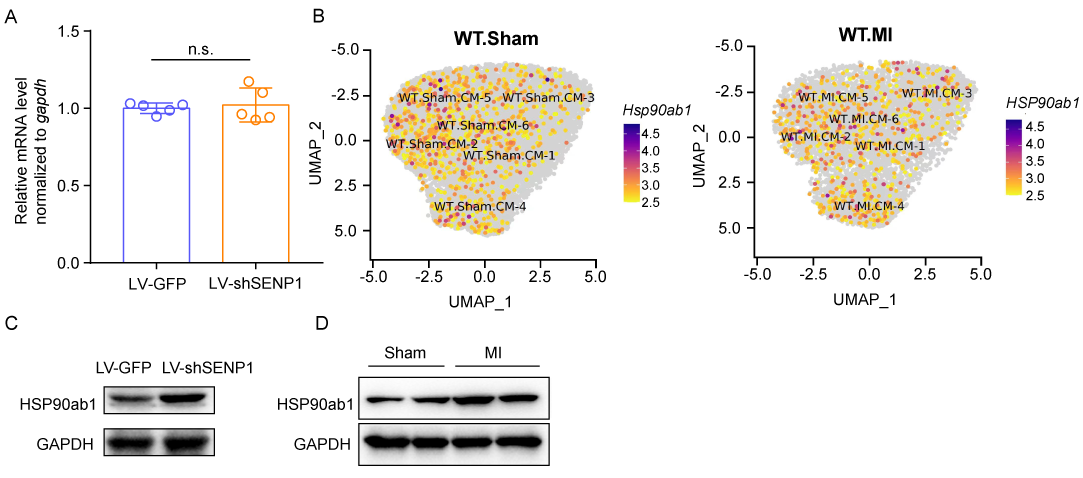
**

**Figure S9. Gene and protein expression pattern of HSP90ab1 (heat shock protein 90 alpha, class B, member 1). (A)** *HSP90ab1* mRNA expression in neonatal rat ventricular myocytes (NRVMs) infected with LV-GFP and LV-shSENP1; n=5 in each group; **(B)** UMAP visualization of clustering revealed *HSP90ab1* expression for each cardiomyocyte subclusters. Data are shown as mean ± SEM. Unpaired t test **(B)**. **(C)** Immunoblots for HSP90ab1 in neonatal rat ventricular myocytes (NRVMs) infected with LV-GFP and LV-shSENP1 for 24 h; **(D)** Immunoblots for HSP90ab1 in primary cardiomyocytes after MI.

**Figure S10**

**
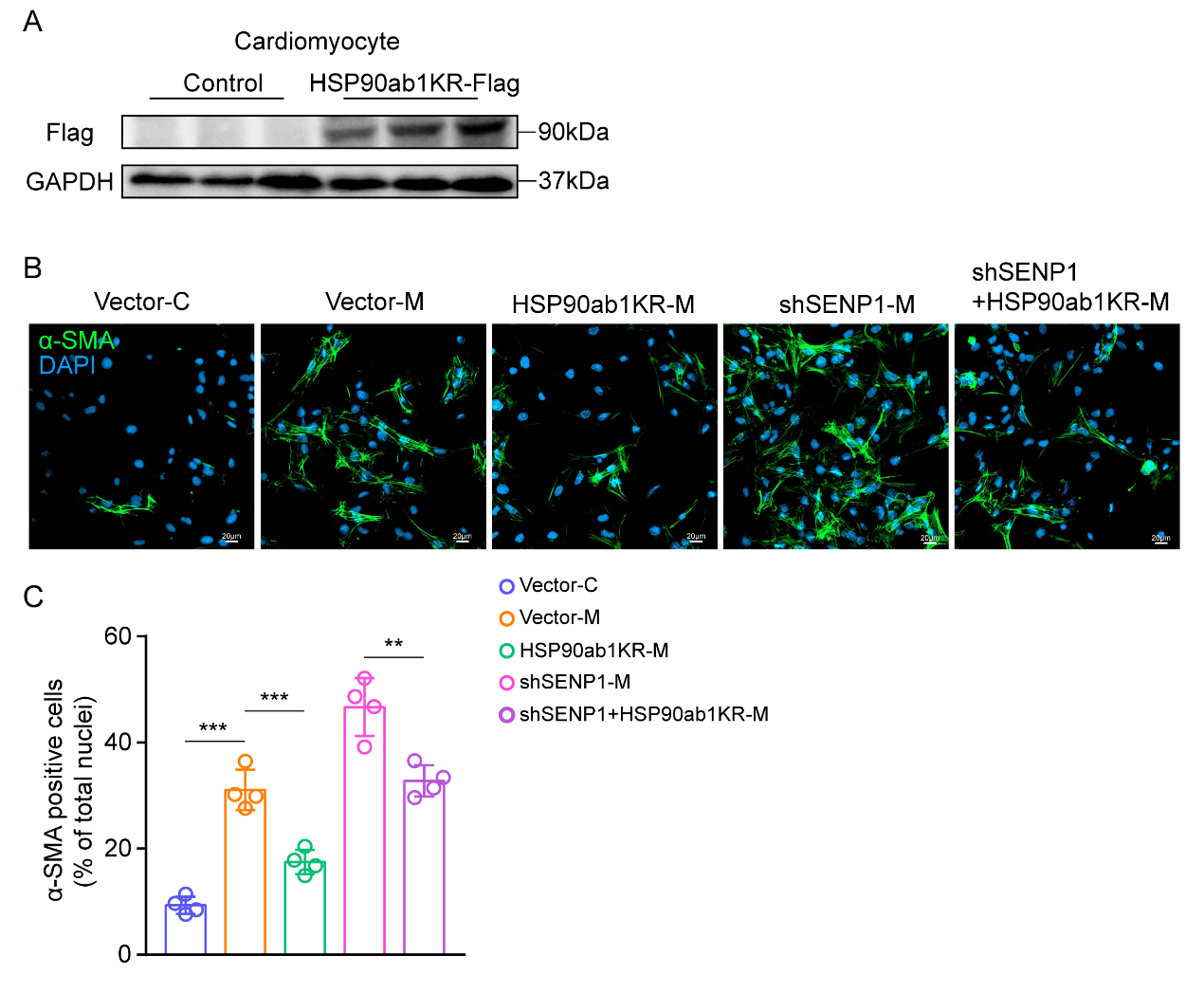
**

**Figure S10. Conditioned medium collected from adeno-associated virus (AAV)-mediated cardiomyocyte-specific HSP90ab1 Lys72 site mutation inhibits the differentiation and proliferation of primary fibroblasts.** **(A)** Immunoblots for Flag in primary cardiomyocytes from CTNT (cardiac troponin T)-Vector and CTNT-HSP90ab1KR-Flag mice; **(B)** Immunofluorescence staining for α-SMA (α-smooth muscle actin)–positive (green) primary fibroblasts; scale bar, 20 μm; (**C)** Quantification of α-SMA positive cells per field; n=4 in each group. Data are shown as mean ± SEM. **p<0.01, and ***p<0.001, using one-way ANOVA followed by Tukey post hoc multiple comparisons test **(C).**

**Table S1** The detailed information about the patients with heart failure

|  | Patient1 | Patient2 | Patient3 | Patient4 |
| --- | --- | --- | --- | --- |
| NYHA | 2 | 2 | 3 | 3 |
| Heart Rate (bpm) | 52 | 80 | 78 | 80 |
| Systolic BP (mmHg) | 126 | 105 | 135 | 134 |
| Diastolic BP (mmHg) | 83 | 76 | 83 | 75 |
| NTproBNP (pg/ml) | 103.5 | 25.84 | 3923 | 1470 |
| hs-TnT (ng/ml) | 0.012 | 0.007 | 0.13 | 0.024 |
| LVEF (%) | 64 | 37 | 30 | 19 |
| BUN (mmol/L) | 4.9 | 5.4 | 6.7 | 5.3 |
| Cr (μmol/L) | 59 | 66 | 103 | 95 |
| Creatinine (mg/dl) | 0.67 | 0.75 | 1.17 | 1.07 |
| Alb (g/L) | 48.7 | 42.5 | 36.9 | 39.3 |
| TBIL (μmol/L) | 8.1 | 7 | 22.5 | 9.2 |
| ALT (μ/L) | 80.6 | 13 | 66.7 | 19.2 |
| AST (μ/L) | 39.5 | 19.9 | 24.9 | 18.3 |

NYHA: New York heart association NYHA classification of heart failure; NTproBNP: N-terminal pro-brain natriuretic peptide; hs-TnT: High-sensitivity cardiac troponin; LVEF: Left ventricular ejection fractions; BUN: Blood urea nitrogen; Cr: Creatinine; Alb: Albumin; TBIL: Total bilirubin; ALT: Alanine aminotransferase; AST: Aspartate aminotransferase.

**Table S2** Upregulated protein information in primary cardiomyocytes from SENP1^flox/flox^Myh6^Cre^ mice vs Myh6^Cre^ mice

| Gene_ID | Myh6^Cre^  mean(n=3) | | | SENP1^flox/flox^Myh6^Cre^  mean(n=3) | log2FoldChange | | *P*.adj | Symbol |
| --- | --- | --- | --- | --- | --- | --- | --- | --- |
| ENSMUSG00000025453 | | 117129.6354 | 17770272.33 | | | 7.271072306 | 1.003E-269 | Nnt |
| ENSMUSG00000000276 | | 233329.2214 | 3268640.917 | | | 3.777266614 | 6.45687E-06 | Dgke |
| ENSMUSG00000057880 | | 137562.0182 | 1383584.833 | | | 3.365905193 | 2.26088E-11 | Abat |
| ENSMUSG00000046598 | | 1015897.375 | 9770913.167 | | | 3.304087324 | 5.14269E-34 | Bdh1 |
| ENSMUSG00000053093 | | 7657918.333 | 38864504 | | | 2.418698866 | 0.000265432 | Myh7 |
| ENSMUSG00000039648 | | 29067.8597 | 156744.7839 | | | 2.417744324 | 0.012300738 | Kyat1 |
| ENSMUSG00000078853 | | 29089.26628 | 134569.7995 | | | 2.217990924 | 2.70304E-06 | Igtp |
| ENSMUSG00000031146 | | 146037.3724 | 626926.5313 | | | 2.108555737 | 0.002693957 | Plp2 |
| ENSMUSG00000057762 | | 146037.3724 | 626926.5313 | | | 2.108555737 | 0.002693957 | Gm6169 |
| ENSMUSG00000026193 | | 97229.53907 | 402656.25 | | | 2.063311203 | 1.31857E-17 | Fn1 |
| ENSMUSG00000041736 | | 92780.78646 | 330008.0938 | | | 1.854922212 | 1.96863E-11 | Tspo |
| ENSMUSG00000020681 | | 25536.17188 | 87830.27474 | | | 1.788896907 | 0.145293641 | Ace |
| ENSMUSG00000001025 | | 77548.73698 | 261014.1771 | | | 1.710207757 | 0.028108454 | S100a6 |
| ENSMUSG00000015085 | | 35917.45052 | 114389.707 | | | 1.655537829 | 0.057046833 | Entpd2 |
| ENSMUSG00000050708 | | 104256.4505 | 312184.0052 | | | 1.59406329 | 0.024730125 | Ftl1 |
| ENSMUSG00000062382 | | 104256.4505 | 312184.0052 | | | 1.59406329 | 0.024730125 | Ftl1-ps1 |
| ENSMUSG00000019278 | | 76223.56901 | 215807.7943 | | | 1.480514194 | 0.2598947 | Dpep1 |
| ENSMUSG00000024659 | | 63342.34115 | 176873.9688 | | | 1.427461554 | 0.171923296 | Anxa1 |
| ENSMUSG00000040249 | | 11959.35807 | 30794.19922 | | | 1.366184811 | 0.162621658 | Lrp1 |
| ENSMUSG00000028001 | | 52840.58984 | 131685.6432 | | | 1.355408191 | 0.162621658 | Fga |
| ENSMUSG00000027204 | | 231013.3542 | 571098.8125 | | | 1.28205228 | 0.019841853 | Fbn1 |
| ENSMUSG00000019929 | | 201691.9115 | 484718.3958 | | | 1.282006927 | 0.002802666 | Dcn |
| ENSMUSG00000018042 | | 1887956.375 | 4490624.5 | | | 1.272434773 | 8.29472E-10 | Cyb5r3 |
| ENSMUSG00000001506 | | 591426.5417 | 1393645.625 | | | 1.227402015 | 0.256326403 | Col1a1 |
| ENSMUSG00000068328 | | 114677.7044 | 262836.4063 | | | 1.22473767 | 0.049242907 | Aup1 |
| ENSMUSG00000028980 | | 15736.56966 | 36647.76693 | | | 1.223799314 | 0.084536498 | H6pd |
| ENSMUSG00000037949 | | 49317.62109 | 113320.6146 | | | 1.209304665 | 0.040730072 | Ano10 |
| ENSMUSG00000020810 | | 86298.46745 | 201122.7344 | | | 1.204512168 | 0.278739859 | Cygb |
| ENSMUSG00000030214 | | 93948.87109 | 212355.4479 | | | 1.203138373 | 0.149854257 | Plbd1 |
| ENSMUSG00000031748 | | 52873.14844 | 123413.3854 | | | 1.19795758 | 0.109111579 | Gnao1 |
| ENSMUSG00000028937 | | 591579.125 | 1314285.292 | | | 1.181364424 | 0.000117199 | Acot7 |
| ENSMUSG00000039048 | | 1995651.458 | 4425707.083 | | | 1.151311454 | 0.002035312 | Foxred1 |
| ENSMUSG00000038776 | | 119644.4557 | 264976.6354 | | | 1.142482276 | 0.009725727 | Ephx1 |
| ENSMUSG00000052605 | | 67224.83203 | 148767.8672 | | | 1.137239665 | 0.012016641 | Isoc2b |
| ENSMUSG00000025287 | | 879360.6667 | 1906224.125 | | | 1.133776485 | 2.39687E-06 | Acot9 |
| ENSMUSG00000003402 | | 191144.8984 | 410356.7604 | | | 1.117637893 | 0.024505508 | Prkcsh |
| ENSMUSG00000039886 | | 60899.84245 | 128785.8411 | | | 1.11077423 | 0.042941913 | Tmem120a |
| ENSMUSG00000019188 | | 173663.9792 | 371304.3229 | | | 1.091240437 | 0.083061399 | H13 |
| ENSMUSG00000026687 | | 73155.33073 | 150524.8281 | | | 1.06711691 | 0.027254011 | Aldh9a1 |
| ENSMUSG00000021388 | | 195520.2526 | 400164.6563 | | | 1.066835023 | 0.079553559 | Aspn |
| ENSMUSG00000042895 | | 474559.0833 | 948204.25 | | | 1.037444184 | 0.039496773 | Abra |
| ENSMUSG00000072640 | | 293685.3906 | 603830.8125 | | | 1.036150319 | 0.264475209 | Lyrm9 |
| ENSMUSG00000024308 | | 84836.16146 | 171420.5286 | | | 1.010155674 | 0.062029742 | Tapbp |
| ENSMUSG00000033557 | | 112705.7786 | 219897.6406 | | | 1.007928575 | 0.046018677 | Fam20b |
| ENSMUSG00000040028 | | 86242.99479 | 176035.4922 | | | 1.006294942 | 0.157664473 | Elavl1 |

**Table S3** Downregulated protein information in primary cardiomyocytes from SENP1^flox/flox^Myh6^Cre^ mice vs Myh6^Cre^ mice

| Gene_ID | Myh6^Cre^  mean(n=3) | SENP1^flox/flox^Myh6^Cre^  mean(n=3) | log2FoldChange | *P*.adj | Symbol |
| --- | --- | --- | --- | --- | --- |
| ENSMUSG00000021390 | 10144  46.422 | 17259.6665 | -5.897565745 | 6.14892E-10 | Ogn |
| ENSMUSG00000071317 | 6976963.438 | 160421.1484 | -5.468695476 | 4.12448E-13 | Bves |
| ENSMUSG00000011148 | 78353.65951 | 3618.835042 | -4.332037734 | 0.002122355 | Adssl1 |
| ENSMUSG00000030470 | 3682556.75 | 189881.9948 | -4.305823638 | 1.71726E-15 | Csrp3 |
| ENSMUSG00000026021 | 1400217.458 | 73005.73438 | -4.287272735 | 8.27014E-34 | Sumo1 |
| ENSMUSG00000001270 | 1196798.688 | 75900.65755 | -3.940178704 | 3.12633E-06 | Ckb |
| ENSMUSG00000079588 | 6973997.51 | 476558.8333 | -3.903649362 | 9.31057E-05 | Tmem182 |
| ENSMUSG00000032332 | 830785 | 59744.91423 | -3.788229242 | 0.000356339 | Col12a1 |
| ENSMUSG00000067338 | 1959724.396 | 184927.5924 | -3.450899339 | 0.038332645 | Tuba3b |
| ENSMUSG00000067702 | 1959724.396 | 184927.5924 | -3.450899339 | 0.038332645 | Tuba3a |
| ENSMUSG00000025040 | 506587.3854 | 56765.94727 | -3.080125252 | 9.19086E-05 | Fundc1 |
| ENSMUSG00000054072 | 326665.3389 | 41500.79167 | -3.051529057 | 0.112546002 | Iigp1 |
| ENSMUSG00000021807 | 89847.75846 | 11542.69173 | -2.925350121 | 0.064327995 | Rtraf |
| ENSMUSG00000033307 | 1361466.958 | 183420.6458 | -2.892600532 | 9.66273E-24 | Mif |
| ENSMUSG00000019987 | 1818048.74 | 240439.8281 | -2.848769146 | 0.002122355 | Arg1 |
| ENSMUSG00000022295 | 156273.7865 | 24042.21159 | -2.687966634 | 0.000555023 | Atp6v1c1 |
| ENSMUSG00000027882 | 94310.58236 | 14902.32113 | -2.653349063 | 0.120390882 | Stxbp3 |
| ENSMUSG00000015749 | 283481.5521 | 48853.41146 | -2.545514925 | 0.012016641 | Anp32e |
| ENSMUSG00000018893 | 20618386.33 | 3555732.083 | -2.54347468 | 0.002041646 | Mb |
| ENSMUSG00000022371 | 85865.44661 | 15645.49902 | -2.446333175 | 0.030916093 | Col14a1 |
| ENSMUSG00000020069 | 163337.6224 | 30273.73568 | -2.420595317 | 0.02002027 | Hnrnph3 |
| ENSMUSG00000046330 | 178778.8021 | 34219.61784 | -2.350477523 | 5.25598E-05 | Rpl37a |
| ENSMUSG00000042717 | 31155.49935 | 6244.312907 | -2.348746989 | 0.023553534 | Ppp1r3a |
| ENSMUSG00000021578 | 1449346.469 | 315697.4557 | -2.28543122 | 0.05070498 | Ccdc127 |
| ENSMUSG00000034187 | 3886278.25 | 757487.1042 | -2.280080962 | 0.002443875 | Nsf |
| ENSMUSG00000051185 | 349745.4792 | 74352.83333 | -2.279892505 | 0.002443875 | Fam174a |
| ENSMUSG00000025190 | 1512597.104 | 320859.7474 | -2.270871978 | 0.018870341 | Got1 |
| ENSMUSG00000008136 | 629914.5625 | 134896.4036 | -2.266979602 | 0.003670892 | Fhl2 |
| ENSMUSG00000017307 | 1497919.604 | 317816.2083 | -2.258985866 | 0.001294464 | Acot8 |
| ENSMUSG00000050174 | 162794.7448 | 32783.16732 | -2.245448503 | 0.003504369 | Nudt6 |
| ENSMUSG00000026817 | 907782.0208 | 202418.6693 | -2.20317281 | 0.012870454 | Ak1 |
| ENSMUSG00000078812 | 163849.8698 | 34567.65951 | -2.195461091 | 0.024396797 | Eif5a |
| ENSMUSG00000028773 | 4963929.167 | 1141515.073 | -2.142846299 | 0.007877985 | Fabp3 |
| ENSMUSG00000030246 | 2557894.75 | 594795.6615 | -2.139668566 | 0.002122355 | Ldhb |
| ENSMUSG00000038383 | 964087.9896 | 228855.1536 | -2.099941661 | 0.030368281 | Pigu |
| ENSMUSG00000046434 | 2438433.375 | 561522.3438 | -2.096276535 | 2.14528E-06 | Hnrnpa1 |
| ENSMUSG00000001175 | 3714549.458 | 903632.5833 | -2.079743911 | 0.034449011 | Calm1 |
| ENSMUSG00000019370 | 3714549.458 | 903632.5833 | -2.079743911 | 0.034449011 | Calm3 |
| ENSMUSG00000036438 | 3714549.458 | 903632.5833 | -2.079743911 | 0.034449011 | Calm2 |
| ENSMUSG00000020277 | 212904.4635 | 50892.52344 | -2.079339898 | 0.002041646 | Pfkl |
| ENSMUSG00000027940 | 2861954.917 | 687004.8542 | -2.052981128 | 0.000422707 | Tpm3 |
| ENSMUSG00000030399 | 32607935.33 | 7783189.167 | -2.048787115 | 0.000427594 | Ckm |
| ENSMUSG00000004558 | 1131129.021 | 284271.4896 | -2.029581337 | 0.006477952 | Ndrg2 |
| ENSMUSG00000025791 | 342525.5469 | 89166.82682 | -1.983798603 | 0.026468599 | Pgm1 |
| ENSMUSG00000069917 | 12826476.67 | 3525878.542 | -1.962476747 | 0.100553584 | Hba-a2 |
| ENSMUSG00000069919 | 12826476.67 | 3525878.542 | -1.962476747 | 0.100553584 | Hba-a1 |
| ENSMUSG00000003559 | 174780.0521 | 44813.42773 | -1.93927563 | 0.010419043 | As3mt |
| ENSMUSG00000056999 | 99582.3099 | 26376.65169 | -1.922356746 | 0.000822646 | Ide |
| ENSMUSG00000052305 | 20177150.33 | 5757657.542 | -1.907399294 | 0.112329369 | Hbb-bs |
| ENSMUSG00000073940 | 20177150.33 | 5757657.542 | -1.907399294 | 0.112329369 | Hbb-bt |
| ENSMUSG00000042828 | 981283.8125 | 273218.0208 | -1.888536076 | 0.008797237 | Trim72 |
| ENSMUSG00000033065 | 805452.4167 | 228049.3646 | -1.828317719 | 0.030108798 | Pfkm |
| ENSMUSG00000005469 | 276555.0729 | 78165 | -1.804643803 | 0.003211968 | Prkaca |
| ENSMUSG00000020572 | 126905.4089 | 39058.32227 | -1.784479707 | 0.282052093 | Nampt |
| ENSMUSG00000018286 | 229958.25 | 69234.27539 | -1.762660658 | 0.049242907 | Psmb6 |
| ENSMUSG00000001891 | 288430.6302 | 86651.48438 | -1.756198339 | 0.010419043 | Ugp2 |
| ENSMUSG00000026755 | 191805.2253 | 57410.91406 | -1.752098405 | 0.115427117 | Arpc5l |
| ENSMUSG00000025068 | 240202512 | 73428453.33 | -1.740637543 | 0.109111579 | Gsto1 |
| ENSMUSG00000024197 | 1879597.854 | 567341.6042 | -1.735278483 | 0.043391743 | Plin3 |
| ENSMUSG00000078427 | 160552.6927 | 50998.29785 | -1.73084943 | 0.234197609 | Sarnp |
| ENSMUSG00000062070 | 2666437.542 | 845863.8958 | -1.69586275 | 0.026468599 | Pgk1 |
| ENSMUSG00000042364 | 83719.33203 | 27232.6263 | -1.683851335 | 0.123689684 | Snx18 |
| ENSMUSG00000021798 | 3533109.167 | 1104298.083 | -1.682666659 | 0.012300738 | Ldb3 |
| ENSMUSG00000062929 | 501725.6563 | 165829.0052 | -1.67697013 | 0.194622249 | Cfl2 |
| ENSMUSG00000015671 | 439841.1484 | 138737.25 | -1.657561654 | 0.126113139 | Psma2 |
| ENSMUSG00000003131 | 74264.53776 | 24372.67253 | -1.645030693 | 0.218769714 | Pafah1b2 |
| ENSMUSG00000028932 | 282998.6042 | 95165.14974 | -1.623402332 | 0.065236313 | Psmc2 |
| ENSMUSG00000028393 | 74970.74479 | 25443 | -1.620883299 | 0.204154511 | Alad |
| ENSMUSG00000032604 | 90883.77214 | 29431.16081 | -1.61747085 | 0.090307105 | Qars |
| ENSMUSG00000061462 | 1101051.708 | 355186.7969 | -1.603994723 | 0.008311506 | Obscn |
| ENSMUSG00000008450 | 127277.8867 | 43591.35026 | -1.592187262 | 0.157664473 | Nutf2 |
| ENSMUSG00000071497 | 127277.8867 | 43591.35026 | -1.592187262 | 0.157664473 | Nutf2-ps1 |
| ENSMUSG00000078348 | 25060.34473 | 8529.126628 | -1.588608205 | 0.204527347 | Sf3b5 |
| ENSMUSG00000020986 | 266882.5417 | 90279.47135 | -1.578341951 | 0.023553534 | Sec23a |
| ENSMUSG00000020435 | 250616.6406 | 84865.17969 | -1.567032802 | 0.095344968 | Osbp2 |
| ENSMUSG00000024687 | 250616.6406 | 84865.17969 | -1.567032802 | 0.095344968 | Osbp |
| ENSMUSG00000031767 | 1279183.938 | 426992.2396 | -1.563805699 | 0.018380146 | Nudt7 |
| ENSMUSG00000031461 | 26491114 | 8839790.667 | -1.551515663 | 0.006491154 | Myom2 |
| ENSMUSG00000028798 | 156392.5781 | 55214.38997 | -1.549741287 | 0.066573843 | Eif3i |
| ENSMUSG00000032306 | 3483066.125 | 1188398.5 | -1.546593398 | 0.03217632 | Mpi |
| ENSMUSG00000026229 | 298293.2656 | 160705.4193 | -1.542173584 | 0.026505949 | Psmd1 |
| ENSMUSG00000002100 | 43558882 | 14810894.67 | -1.537665224 | 0.009852807 | Mybpc3 |
| ENSMUSG00000060843 | 228172.5521 | 77532.97526 | -1.529854732 | 0.012456556 | Ctnna3 |
| ENSMUSG00000028024 | 339150.125 | 116323.4271 | -1.521899625 | 0.130412778 | Enpep |
| ENSMUSG00000024049 | 13742310.67 | 4704384.75 | -1.519961626 | 0.004800458 | Myom1 |
| ENSMUSG00000026245 | 41034.6556 | 14366.63639 | -1.517779018 | 0.262612681 | Farsb |
| ENSMUSG00000005779 | 278918.5833 | 97090.06771 | -1.517714107 | 0.027133063 | Psmb4 |
| ENSMUSG00000071644 | 122594.3646 | 44295.35091 | -1.503238004 | 0.118521699 | Eef1g |
| ENSMUSG00000030917 | 170880.8255 | 61980.29753 | -1.487995597 | 0.133379255 | Tmem159 |
| ENSMUSG00000042010 | 60859.17578 | 21339.22721 | -1.484913268 | 0.230814186 | Acacb |
| ENSMUSG00000020386 | 127020.4089 | 46348.06315 | -1.477787342 | 0.065099827 | Sar1b |
| ENSMUSG00000020321 | 3853564.667 | 1398898.333 | -1.474025305 | 0.002875342 | Mdh1 |
| ENSMUSG00000074652 | 631174.1458 | 230894.5078 | -1.468307258 | 0.016629701 | Myh7b |
| ENSMUSG00000030847 | 4124338.917 | 1438760.729 | -1.457966706 | 0.055321528 | Bag3 |
| ENSMUSG00000021660 | 336769.9792 | 205700.1354 | -1.455431533 | 0.012551954 | Btf3 |
| ENSMUSG00000035337 | 83403.46875 | 31307.83268 | -1.437275034 | 0.079553559 | Uchl4 |
| ENSMUSG00000025607 | 41773.74219 | 15562.65479 | -1.435309189 | 0.25779234 | Copg2 |
| ENSMUSG00000030058 | 41773.74219 | 15562.65479 | -1.435309189 | 0.25779234 | Copg1 |
| ENSMUSG00000015714 | 74012.10807 | 27239.48438 | -1.420732986 | 0.106530077 | Cers2 |
| ENSMUSG00000020475 | 1180858.833 | 448634.2969 | -1.416446268 | 0.034449011 | Pgam2 |
| ENSMUSG00000030695 | 3986491.167 | 1539390.479 | -1.412486492 | 0.073280961 | Aldoa |
| ENSMUSG00000068882 | 485254.4427 | 175614.1615 | -1.410001427 | 0.163458012 | Ssb |
| ENSMUSG00000032648 | 2682715.667 | 992837.9792 | -1.402239775 | 0.095629079 | Pygm |
| ENSMUSG00000051695 | 297181.1198 | 115501.819 | -1.401356654 | 0.062080031 | Pcbp1 |
| ENSMUSG00000026750 | 279908.6302 | 105688.1068 | -1.3990059 | 0.006491154 | Psmb7 |
| ENSMUSG00000019055 | 221065.3672 | 87068.85286 | -1.393028492 | 0.216611843 | Plod1 |
| ENSMUSG00000057113 | 343180.2188 | 134994.9941 | -1.389066427 | 0.238418436 | Npm1 |
| ENSMUSG00000117924 | 184154.3359 | 68224.28646 | -1.387111105 | 0.172393811 | Tmem223 |
| ENSMUSG00000030785 | 1864987.271 | 743232.974 | -1.383948602 | 0.181692637 | Cox6a2 |
| ENSMUSG00000069769 | 220516.7917 | 85800.52995 | -1.375585996 | 0.023553534 | Msi2 |
| ENSMUSG00000050965 | 460821.4583 | 179789.388 | -1.368505259 | 0.08704639 | Prkca |
| ENSMUSG00000063457 | 824216.5417 | 311517.4219 | -1.357430402 | 0.012551954 | Rps15 |
| ENSMUSG00000033860 | 405349.6771 | 162995.9948 | -1.34943313 | 0.252607874 | Fgg |
| ENSMUSG00000012848 | 1235006.75 | 475917.5729 | -1.33138169 | 0.004713894 | Rps5 |
| ENSMUSG00000018845 | 1381682.208 | 537191.7083 | -1.329073824 | 0.021959427 | Unc45b |
| ENSMUSG00000039103 | 74316.63411 | 30195.125 | -1.321026724 | 0.160851201 | Nexn |
| ENSMUSG00000039001 | 505984.0208 | 202941.5208 | -1.316404964 | 0.030393585 | Rps21 |
| ENSMUSG00000073557 | 152189.0833 | 60978.26302 | -1.316314407 | 0.079553559 | Ppp1r12b |
| ENSMUSG00000054455 | 599714.7188 | 244330.6354 | -1.316084208 | 0.027609253 | Vapb |
| ENSMUSG00000031807 | 224435.5208 | 91444.13021 | -1.315291305 | 0.126244626 | Pgls |
| ENSMUSG00000032959 | 447351.125 | 187648.2357 | -1.31044569 | 0.175164445 | Pebp1 |
| ENSMUSG00000020745 | 164602.6198 | 67624.78385 | -1.307187647 | 0.187651049 | Pafah1b1 |
| ENSMUSG00000034254 | 66548.88346 | 26115.34245 | -1.305478171 | 0.275252958 | Agpat1 |
| ENSMUSG00000057322 | 142334.7031 | 57068.83529 | -1.302796147 | 0.197437424 | Rpl38 |
| ENSMUSG00000060600 | 5723089.75 | 2314186.625 | -1.295924571 | 0.006110782 | Eno3 |
| ENSMUSG00000024236 | 277610.6563 | 112717.8099 | -1.279528432 | 0.02628147 | Svil |
| ENSMUSG00000001642 | 1183672.208 | 500960.7604 | -1.275834537 | 0.117101557 | Akr1b3 |
| ENSMUSG00000029780 | 315388.9531 | 127774 | -1.269766758 | 0.146360331 | Nt5c3 |
| ENSMUSG00000029338 | 141335.4401 | 58111.96484 | -1.26600926 | 0.010641194 | Antxr2 |
| ENSMUSG00000033059 | 73642018.67 | 30203458 | -1.265280032 | 0.109111579 | Pygb |
| ENSMUSG00000017390 | 1262605.083 | 549931.1354 | -1.258766326 | 0.143928446 | Aldoc |
| ENSMUSG00000023277 | 296697.5911 | 127764.263 | -1.23639238 | 0.2598947 | Twf2 |
| ENSMUSG00000047221 | 158026.8021 | 65314.4349 | -1.218137624 | 0.079553559 | Fam185a |
| ENSMUSG00000026202 | 392785.0104 | 170284.6406 | -1.208018055 | 0.045407523 | Tuba4a |
| ENSMUSG00000059040 | 6770514.167 | 2951249.5 | -1.198211458 | 0.019841853 | Eno1b |
| ENSMUSG00000063524 | 6770514.167 | 2951249.5 | -1.198211458 | 0.019841853 | Eno1 |
| ENSMUSG00000020949 | 209442.2161 | 94401.77344 | -1.193009269 | 0.275252958 | Fkbp3 |
| ENSMUSG00000047215 | 660267.5625 | 292247.0729 | -1.191486143 | 0.097015505 | Rpl9 |
| ENSMUSG00000062456 | 660267.5625 | 292247.0729 | -1.191486143 | 0.097015505 | Rpl9-ps6 |
| ENSMUSG00000022856 | 138478.7057 | 60036.09115 | -1.174834832 | 0.080779842 | Tmem41a |
| ENSMUSG00000027012 | 140471.4688 | 63415.26953 | -1.164715169 | 0.034449011 | Dync1i2 |
| ENSMUSG00000026701 | 290641.5104 | 132496.3464 | -1.164403597 | 0.157664473 | Prdx6 |
| ENSMUSG00000025104 | 105034.4401 | 46197.59766 | -1.162611217 | 0.047710399 | Hdgfl3 |
| ENSMUSG00000032294 | 2035579.333 | 927701.7813 | -1.162510685 | 0.09762733 | Pkm |
| ENSMUSG00000020869 | 302138.776 | 133643.0625 | -1.16062677 | 0.234971022 | Lrrc59 |
| ENSMUSG00000011257 | 537746.4896 | 237843.1042 | -1.145336504 | 0.079553559 | Pabpc4 |
| ENSMUSG00000032216 | 73459.86198 | 33464.40365 | -1.141912787 | 0.063658136 | Nedd4 |
| ENSMUSG00000068184 | 1089361.104 | 483367.9375 | -1.131937042 | 0.028845041 | Ndufaf2 |
| ENSMUSG00000023456 | 2145708.667 | 1000660 | -1.110045215 | 0.100553584 | Tpi1 |
| ENSMUSG00000005474 | 374667.9896 | 177663.9349 | -1.099415226 | 0.151765304 | Myl10 |
| ENSMUSG00000029759 | 354408.2083 | 164100.6094 | -1.09010179 | 0.030108798 | Pon3 |
| ENSMUSG00000068749 | 177097.1927 | 83080.16927 | -1.085179218 | 0.083061399 | Psma5 |
| ENSMUSG00000028273 | 1258189.292 | 590061.7292 | -1.080701763 | 0.002146936 | Pdlim5 |
| ENSMUSG00000020483 | 187266.1563 | 89537.66797 | -1.076798065 | 0.160851201 | Dynll2 |
| ENSMUSG00000002500 | 349930.1094 | 161497.5521 | -1.076077448 | 0.187651049 | Rpl3l |
| ENSMUSG00000061477 | 1056632.083 | 493754.0938 | -1.074289913 | 0.042415099 | Rps7 |
| ENSMUSG00000035441 | 94334.79948 | 43760.74349 | -1.073966353 | 0.160851201 | Myo1d |
| ENSMUSG00000018509 | 578881.0313 | 264256.0156 | -1.072482072 | 0.221336896 | Cenpv |
| ENSMUSG00000021147 | 235531.1198 | 111561.6146 | -1.071973717 | 0.051417886 | Wdr37 |
| ENSMUSG00000023088 | 251391.7188 | 115616.4818 | -1.069216094 | 0.131780664 | Abcc1 |
| ENSMUSG00000000631 | 1547272.417 | 736245.1458 | -1.055599971 | 0.000555023 | Myo18a |
| ENSMUSG00000022853 | 367153.1667 | 171883.5729 | -1.052386181 | 0.019582106 | Ehhadh |
| ENSMUSG00000020720 | 506206.3854 | 241601.1771 | -1.04699153 | 0.026468599 | Psmd12 |
| ENSMUSG00000003873 | 377878.7969 | 185301.5729 | -1.03980926 | 0.229232726 | Bax |
| ENSMUSG00000057666 | 7164808 | 3564241.5 | -1.036774345 | 0.15955766 | Gapdh |
| ENSMUSG00000097148 | 7164808 | 3564241.5 | -1.036774345 | 0.15955766 | Gm3839 |
| ENSMUSG00000017817 | 274337.2656 | 132985.2578 | -1.030012426 | 0.079442498 | Jph2 |
| ENSMUSG00000016833 | 109259.7188 | 53588.35286 | -1.021163956 | 0.2598947 | Mrps18c |
| ENSMUSG00000030603 | 148483.5573 | 73811.42708 | -1.003073863 | 0.026468599 | Psmc4 |

**Table S4** Different databases identify transcription factors regulating Fn

| Human (GTRD) | | | | Mouse (GTRD) | | | hTFtarget | HumanTFDB | MERGE |
| --- | --- | --- | --- | --- | --- | --- | --- | --- | --- |
| APC | NKX3-1 | DNMT3B | ZNF148 | Nr3c1 | LMNB1 | PPARD | SPL1 | BTAF1 | HDAC2 |
| SAP130 | SIX2 | OGG1 | EGR | Etv4 | SREBF1 | TFAP2C | ATF2 | HDAC2 | REST |
| RUVBL1 | REST | MAFG | MXD3 | Kdm5b | CEBPE | GTF2B | BATF | REST | FOXA2 |
| SMARCB1 | CTBP1 | BRD1 | CRY1 | Sp3 | ATOH1 | TAF10 | BCL11A | MYB | SPI1 |
| BRCA2 | NCOA1 | MAFF | OTX2 | Spi1 | PPARA | RAD21 | BCL6 | FOXA2 | EP300 |
| CEBPB | IRF4 | MIER3 | ZNF384 | Srsf2 | DPPA4 | ATF7 | BRD4 | SPI1 | BRD4 |
| SMAD3 | INO80 | TFAP2A | GATA2 | Zfp874a | OTX2 | MECP2 | CDK9 | ORC1 | SRF |
| ESR2 | NCOR1 | NCAPH2 | ZBTB20 | Dr1 | MED24 | NIPBL | CEBPA | EP300 | SMAD1 |
| MSC | RARG | LHX9 | PAX3 | Pbx1 | JARID2 | ARNT2 | CREB1 | BRD4 | JUND |
| STAG2 | SP4 | NFATC3 | TRIM28 | Isx | NRF1 | NEUROD2 | E2F1 | SP4 | CDK8 |
| ME1 | NOTCH1 | NR2C2 | HLF | Max | TRIM28 | KDM2A | EP300 | SRF | SMARCA4 |
| BPTF | SMAD2 | NELFE | VEZF1 | Epop | ESR2 | ZFP874B | ERG | SMAD1 | STAT3 |
| FOSB | NR1H3 | ESR1 | H2AFZ | RTF1 | CLOCK | SIX4 | GATA1 | SP2 | IRF1 |
| PRDM10 | ETV4 | PGR | ZNF398 | ELL3 | SALL4 | ZBTB2 | TRF1 | TCF4 | SP1 |
| PRDM9 | HES1 | ZSCAN9 | SP5 | SOX11 | FOXH1 | PCGF2 | MAZ | FOXG1 |  |
| FXR2 | NFIL3 | RB1 | NCOR2 | GATA1 | BRD2 | ESRRB | MED1 | ZBTB7B |  |
| RAD21 | DDIT3 | HOXB13 | PAX5 | USP16 | KLF3 | TBX3 | MYB | JUND |  |
| ZEB1 | SMAD1 | ZNF350 | TLE3 | PRDM13 | OLIG2 | TBX5 | PAX5 | POU5F1 |  |
| TRIM22 | FUS | KDM6A | GATA3 | ETV6 | CDK7 | ZFP953 | PBX3 | CDK8 |  |
| MGA | HIRA | JUND | MAZ | PKNOX1 | NKX2-5 | MEN1 | RUNX1 | SMARCA4 |  |
| PEX2 | PHF8 | DRAP1 | PKNOX1 | NR6A1 | NR1H3 | PHF5A | RUNX1T1 | RFX5 |  |
| AGO2 | NCOA3 | ATF7 | TRIM24 | GFI1B | NKX2-2 | CRTC2 | STAT1 | SP3 |  |
| TBX5 | SKI | ZEB2 | HDGFL3 | SOX3 | SUZ12 | PAX3 | STAT5B | STAT3 |  |
| TEAD3 | ZNF217 | ZSCAN4 | E2F6 | SP1 | NR1D1 | RAG1 | TBX21 | SPI4 |  |
| ASF1A | MIXL1 | TAL1 | AR | MSX1 | ZFP808 | PITX1 | TCF7L2 | CF4 |  |
| PBX3 | CDKN1B | LMNB1 | RARA | LHX2 | CHD4 | ZFP938 | VDR | NOTCH1 |  |
| PTBP1 | CHD7 | PATZ1 | RELB | FOXP3 | SALL1 | NPAS3 | ETS1 | IRF1 |  |
| ZNF316 | TEAD4 | PHOX2B | GLIS1 | RFX3 | PHRF1 | MAFG | ARID3A | SP1 |  |
| GRHL1 | WDR5 | HAND2 | ME3 | CHAF1B | THRA | MAFF | CBX2 |  |  |
| MBD1 | TARDBP | DACH1 | HEY1 | FEZF2 | MAGIL | MSL2 | CEBPB |  |  |
| UBP1 | PBX4 | SPDEF | ZNF792 | TET3 | YAP1 | POU5F1 | CEBPD |  |  |
| RFX5 | AHR | TFE3 | GATA1 | E2F3 | KLF5 | GATA3 | E2F4 |  |  |
| PRDM6 | NRF1 | GTF3C2 | FEZF1 | RUNX2 | TCF4 | AR | E2F6 |  |  |
| HNRNPH1 | ERCC2 | SCRT2 | TCF7L2 | BACH1 | KMT2A | TBL1X | EGR1 |  |  |
| MNT | NR2F6 | ETV6 | NONO | STAT6 | BCL11B | CREB3L2 | FOS |  |  |
| NFIA | NR2F1 | MYOG | POU2F1 | KMT2D | RBBP4 | NFIA | FOSL1 |  |  |
| HNF1A | PADI2 | MYOD1 | TCF7 | ZFP973 | EOMES | SIRT6 | GATA2 |  |  |
| MAFK | FOXH1 | CAT | MAF | GM14406 | NCOR1 | UTF1 | HDAC1 |  |  |
| NFKB1 | HOXA9 | SRSF3 | TFAP4 | NKX2-1 | CEBPB | HXOB4 | JUN |  |  |
| DTL | DMAP1 | CREB3L1 | CTCF | MEIS1 | SMARCAD1 | FOXA2 | JUND |  |  |
| U2AF2 | ZNF554 | NOTCH3 | FANCD2 | KDM2B | RELA | FOXA1 | KDM5B |  |  |
| STAT2 | HIF1A | PPARD | CEBPD | CDK8 | MAFK | CHD2 | MAX |  |  |
| NFYC | HNRNPK | RUNX1T1 | ZIC5 | MSL1 | NRDC | CAPG | MYC |  |  |
| RUNX2 | EN1 | MBD4 | CEBPA | JUND | PRDM14 | DUX | NR2F2 |  |  |
| AGO1 | RBM39 | WT1 | AMH | IRF4 | MYOG | KMT2C | PHF8 |  |  |
| LEO1 | EBNA1BP2 | NR5A2 | FOXM1 | BRD4 | ELF5 | ZFP217 | PML |  |  |
| TBX21 | TGIF2 | NFIC | SNAI2 | DPEP2 | PURB | MEF2C | POLR2A |  |  |
| LHX2 | SCRT1 | CUL4A | HNRNPL | MEF2A | RUNX1 | HOXA13 | RCOR1 |  |  |
| RING1 | FOXO3 | RORC | CASZ1 | CBX2 | LMO2 | MYCN | SMAD1 |  |  |
| ZNF157 | ZNF35 | KAT7 | MCM7 | LHX3 | NFE2L2 | ZFP429 | SMARCA4 |  |  |
| ZNF48 | ZNF652 | SP2 | FGFR1 | ETV5 | DPY30 | PHF6 | SUZ12 |  |  |
| BRD4 | GRHL3 | ESCO2 | E2F1 | ISL1 | RARA | RAD51 | TAL1 |  |  |
| RBL2 | MBL2 | MAX | STAT1 | ZNF281 | FLOT1 | DPF2 | TEAD4 |  |  |
| BHLHE40 | SUPT6H | CHD1 | JUNB | POU3F1 | DMC1 | SMYD3 | TRIM28 |  |  |
| CREM | FLI1 | SNRNP70 | ZNF3 | NKX3-2 | KLF4 | ARD3A | UBTF |  |  |
| HDAC2 | HNF1B | SREBF2 | STAT5A | THAP1 | SIN3A | SMAD2 | USF1 |  |  |
| EHF | CBX3 | FOXO1 | STAT6 | TET2 | ATF3 | MED12 | YY1 |  |  |
| ZBTB7A | EOMES | STAT5B | KLF9 | ASCL2 | CREB1 | DNMT3A | ZBTB7A |  |  |
| RBPJ | LMNA | MEF2A | HEXIM1 | PAX7 | TFAP4 | CEBPD | ZNF384 |  |  |
| HOXB8 | EBP | KMT2C | U2AF1 | PWWP2B | FOXK1 | DNMT3B | CREBBP |  |  |
| UBTF | PCBP2 | NIPBL | HMG20B | GM14419 | SMARCC1 | AFF4 | FOSL2 |  |  |
| LCORL | ZFP64 | KMT2A | KLF5 | CTR9 | SOX4 | CRX | FOXM1 |  |  |
| INTS12 | PCBP1 | ARID2 | IKZF5 | NUP98 | SOX9 | MTA2 | GATA3 |  |  |
| EZH2 | SRSF4 | BCL3 | FOXA3 | CTCF | IRF8 | E2F4 | MEF2A |  |  |
| SOX13 | RBBP4 | RNF2 | FOXA1 | RSF1 | SNAI2 | NR4A1 | NFIC |  |  |
| TP53BP1 | ESRRA | INTS11 | KAT8 | PCGF3 | PTF1A | NEUROD1 | REST |  |  |
| MEIS2 | VDR | ZNF580 | TCF12 | TFCP2L1 | SRF | STAT1 | RXRA |  |  |
| L3MBTL4 | HOXC5 | INSR | SSRP1 | SMARCA4 | SMAD4 | NFYA | SIN3A |  |  |
| BRCA1 | RUNX1 | TP53 | ASH2L | STAG2 | AURKB | KDM4C | TAF1 |  |  |
| EHMT2 | SLC30A9 | MYF5 | ORC2 | HDAC2 | MORC3 | SETDB1 | TCF12 |  |  |
| TFAP2C | ONECUT2 | TRPS1 | HHEX | ATF | VDR | NCAPD3 | AR |  |  |
| IKZF2 | EPAS1 | ZNF317 | CCAR2 | XBP1 | REST | STAT3 | ARNT |  |  |
| THRB | ELF3 | SPI1 | HDAC3 | YY1 | BTAF1 | DPPA2 | CTCF |  |  |
| CHD4 | EP400 | NFE2L2 | L3MBTL2 | SP9 | PRDM15 | PRDM1 | ESR1 |  |  |
| BATF3 | KDM5A | ZHX2 | HBP1 | RNF2 | ZFP982 | IKZF1 | FOXA1 |  |  |
| CHD8 | OLIG2 | HNRNPC | PARP1 | CCNY | ONECUT2 | GPS2 | GRHL2 |  |  |
| NFKBIZ | EMSY | ZBTB24 | KLF16 | TP53 | ANPEP | RBFOX2 | HDAC2 |  |  |
| ZBTB26 | SRC | ERF | IKZF1 | CDK9 | MED26 | SSRP1 | PGR |  |  |
| GABPA | ZSCAN5A | ETV1 | MTA2 | PCGF6 | PPARGC1A | NELFE | RAD21 |  |  |
| GABPB1 | MTOR | PRMT1 | XRCC4 | CTCFL | RUNX3 | HOXA9 | STAG1 |  |  |
| FOXA2 | KDM3A | MYC | KDM1B | STRA8 | PROP1 | DPEP3 | STAT3 |  |  |
| NR3C1 | THAP11 | IVNS1ABP | ERCC3 | SIX2 | KAT8 | SATB1 | TFAP2C |  |  |
| KDM5B | HMGXB4 | ZSCAN2 | ZBTB14 | FOXO1 | GTF3C1 | KDM3A | FOXA2 |  |  |
| TEAD1 | ARID4B | PRPF4 | BDP1 | RXRA | ETS1 | RARB | NR3C1 |  |  |
| BRD9 | RUNX3 | FOS | MYCN | UTP6 | ZBTB17 | TEX10 | RELA |  |  |
| RCOR1 | EED | SOX2 | HDAC6 | RFX1 | TET1 | ATF4 | TFAP2A |  |  |
| SOX17 | YAP1 | HNRNPLL | STAT4 | GATA4 | AHR | NFATC1 | ATF3 |  |  |
| KDM7A | GRHL2 | ASCL1 | USF2 | SUPT16H | NPAS4 | CEBPA | CBX3 |  |  |
| FOSL2 | ZNF341 | ZNF467 | PRDM1 | PPARG | ZFP759 | BAZ1B | CDK8 |  |  |
| FOSL1 | EBF1 | TOP1 | JMJD6 | TLE3 | HIC2 | BCL6 | FOXO3 |  |  |
| IRF2 | EGR3 | POU5F1 | BCL6 | ZFP457 | ABCC9 | CEBPG | KMT2B |  |  |
| TSC22D4 | DAXX | RBM25 | CEBPG | NUP153 | NKX3-1 | CXXC5 | REPIN1 |  |  |
| KLF15 | CBX1 | CDX2 | USF1 | HNF4A | TWIST2 | JUNB | SP1 |  |  |
| TCF4 | ZBED5 | E2F4 | ERCC6 | TEAD4 | ESR1 | TEAD1 | SRF |  |  |
| GATAD1 | RXRA | ZNF143 | NFATC1 | ERF | RORA | KAT5 | TCF21 |  |  |
| SUZ12 | GATAD2B | PBXIP1 | CBX8 | ZFP384 | SNAI1 | NR1D2 | CTBP2 |  |  |
| ZNF701 | HNF4A | KLF6 | RFX1 | ELK1 | MTA1 | ZFP992 | EOMES |  |  |
| ZC3H8 | CREBBP | MED12 | SMC3 | PRDM16 | KDM6A | POU2F3 | FOXH1 |  |  |
| ZBTB33 | SMARCA5 | EGR2 | ZBTB40 | HEY1 | RORC | INSM1 | GATA4 |  |  |
| ARNTL | FIP1L1 | ZBTB48 | NFYA | JUN | RAD23B | NR1H4 | KDM4A |  |  |
| NSD2 | CTBP2 | NR2F2 | ZIC2 | IRF9 | HEY2 | TBP | MXI1 |  |  |
| EP300 | HDAC1 | TET3 | PMEPA1 | HDAC1 | TBPL1 | EBF1 | NANOG |  |  |
| OVOL2 | ELF1 | ARID3A | HNF4G | GATA2 | NELFB | POU3F2 | OTX2 |  |  |
| RBFOX2 | HOMEZ | AFF1 | SUPT16H | PEX2 | EP300 | FOSL2 | SAP30 |  |  |
| ETV2 | SRF | NANOG | CBX2 | HNF1B | HAND2 | RAG2 | SP4 |  |  |
| NFKB2 | RELA | ZBTB21 | GCM2 | NOTCH1 | TCF7L1 | E2F1 | LHX2 |  |  |
| SOX8 | MLX | EWSR1 | ARID1B | ZFAT | NFIB | BHLHE40 | PAX6 |  |  |
| ZNF449 | SRSF1 | GLIS2 | EGR1 | EZH2 | TERF2IP | STAT5B | GTF2I |  |  |
| SETDB1 | PROX1 | AFF4 | ETV5 | HCFC1 | FOSB | STAT5A | KLF4 |  |  |
| SFPQ | DPF2 | UBN1 | ZNF444 | SUPT5H | ERG | BMI1 | SPI1 |  |  |
| BRD2 | KDM5C | GFI1 | KDM1A | ELOB | MYOD1 | NR1I2 | BMI1 |  |  |
| E2F8 | APOBEC3B | CREB5 | SOX5 | FLI1 | DMRT1 | ZFP709 | BRD2 |  |  |
| SMARCA2 | RFXANK | HBZ | GATAD2A | ZFPM1 | NCOA3 |  | NCOR1 |  |  |
| CRTC2 | MLLT1 | CDK8 | TBP | SMC3 | MED23 |  | RNF2 |  |  |
| ZNF664 | MECOM | ZBTB25 | ZGPAT | TCF3 | NCAPH2 |  | RYBP |  |  |
| CTNNB1 | HMG20A | PALB2 | SOX4 | LDB1 | ARID1A |  | ZNF263 |  |  |
| CTCFL | FOXK2 | STAT3 | ARID1A | DNMT1 | NKX6-1 |  | ELF1 |  |  |
| SMARCA4 | ZXDC | PRDM14 |  | SFMBT2 | ZEB1 |  | HNF4A |  |  |
| BMI1 | F10 | TAF15 |  | ZIC3 | ASXL1 |  | HNF4G |  |  |
| ZNF263 | NEUROD1 | SMARCE1 |  | ZIC2 | TCF12 |  | MAFF |  |  |
| SIN3A | SRCAP | CNOT3 |  | KMT2B | HDGFL2 |  | MBD4 |  |  |
| CBFA2T3 | ETS1 | ATF2 |  | EBF2 | MYC |  | MYBL2 |  |  |
| PTEN | LYL1 | SIN3B |  | OGDH | PROX1 |  | RFX5 |  |  |
| RBBP5 | ZBTB16 | ATF1 |  | ATRX | SOX2 |  | SMAD3 |  |  |
| BRD3 | TET2 | ATF4 |  | STAG1 | CBFB |  | ASCL1 |  |  |
| RXRB | HNRNPUL1 | ATF3 |  | NR5A2 | HOXB5 |  | CHD1 |  |  |
| KLF4 | RAD51 | E2F7 |  | ZFP985 | ZFP595 |  | E2F7 |  |  |
| HMGB2 | EBF3 | KDM4A |  | VSX2 | NCOA2 |  | GTF2B |  |  |
| YY1 | ZNF644 | CDK9 |  | SPEN | SHOX2 |  | LMNB1 |  |  |
| ZNF639 | GATA4 | RBM22 |  | LEO1 | DLX5 |  | MAFK |  |  |
| KLF11 | BICRA | BRD7 |  | GLI2 | ZFP57 |  | NKX2-1 |  |  |
| ELK3 | MLXIP | MECP2 |  | SETD11 | PHF21B |  | RB1 |  |  |
| JUN | SP140 | DCP1A |  | SINHCAF | SAP18 |  | RBL2 |  |  |
| CENPA | NKX2-1 | IRF9 |  | SP7 | CBX3 |  | SMAD4 |  |  |
| p65 | ZBTB17 | TP63 |  | NEUROG2 | BBX |  | FLI1 |  |  |
| DUX4 | GLIS3 | SATB1 |  | UBN2 | LYL1 |  | EWSR1 |  |  |
| PPARG | RAG2 | NUP153 |  | NCOR2 | SMAD1 |  | WDR5 |  |  |
| SMARCC1 | SNAPC4 | ARNT |  | IRF3 | NANOG |  | MYOD1 |  |  |
| ZNF366 | FOXP1 | SIRT6 |  | ETS2 | SIM2 |  | KDM4C |  |  |
| ZNF770 | CDK2 | PDX1 |  | RBPJ | AFF3 |  | DDX5 |  |  |
| KMT2B | TCF7L1 | DEAF1 |  | CDX2 | FOXF1 |  | DUX4 |  |  |
| XRCC5 | NFE2 | HIF3A |  | ESRRA | ZFP935 |  | KLF9 |  |  |
| HSF1 | SS18 | NUP98 |  | CBX7 | GFI1 |  | MITF |  |  |
| ZNF614 | HMBOX1 | DMC1 |  | NELFA | PLAGL1 |  | NFATC1 |  |  |
| ZNF395 | SMC1A | PHF5A |  | INO80 | LIN28A |  | NRIP1 |  |  |
| MBTPS2 | TAF1 | GMEB2 |  | SOX17 | ELL2 |  | ONECUT1 |  |  |
| GATA6 | IRF1 | CBFA2T2 |  | ACAA2 | SMARCA5 |  | SOX2 |  |  |
| MEIS3P1 | ZSCAN5DP | ASCL2 |  | ARNTL | ZNF24 |  | TP53 |  |  |
| GTF2F1 | ZFP1 | SMAD4 |  | TP63 | SUPT6H |  | IRF1 |  |  |
| MITF | MBD2 | SRSF9 |  | NFIL3 | MED1 |  | MYH11 |  |  |
| NEUROG2 | SUPT5H | TCF21 |  | HDAC3 | PBEM1 |  | ETV4 |  |  |
| UPF1 | RERE | KDM3B |  | KDM1A | SMC1A |  | FOXP1 |  |  |
| ZKSCAN8 | CREB1 | MYB |  | PAX5 | FOXL2 |  | RUNX2 |  |  |
| ZMYM3 | ZNF592 | MYBL2 |  | SMAD3 | TAL1 |  | GATA6 |  |  |
| SRSF7 | MED16 | GREB1 |  | ONECUT1 | ZFP92 |  | ELK3 |  |  |
| KLF10 | CLOCK | MXD4 |  | TOP2B | SIRT1 |  |  |  |  |
| BCL11A | MIER2 | BATF |  | BRCA1 | IRF1 |  |  |  |  |
| MYRF | ZNF205 | KDM2B |  | CREBBP | TBR1 |  |  |  |  |
| SP1 | ZNF511 | TWIST1 |  | HNF1A | USF1 |  |  |  |  |

**Table S5** Anti-SENP1/SUMO pull-down protein was identified in primary cardiomyocytes by mass spectrometry(MI vs Sham Fold change＞4)

| Accession | Coverage [%] | Peptides | PSMs | Unique Peptides | AAs | MW [kDa] | calc. pI | Abundances(Sham) | Abundances(MI) |
| --- | --- | --- | --- | --- | --- | --- | --- | --- | --- |
| P01872 | 33 | 12 | 15 | 12 | 454 | 49.9 | 7.01 | 3233828.25 | 36936577.1 |
| Q3THU8 | 8 | 3 | 5 | 3 | 357 | 39.6 | 9.19 | 1123473.625 | 12498444.11 |
| Q921I1 | 48 | 29 | 41 | 29 | 697 | 76.7 | 7.18 | 10381829.22 | 108589733.7 |
| P07724 | 74 | 36 | 214 | 36 | 608 | 68.6 | 6.07 | 388071162.2 | 3331455843 |
| A2ACH6 | 13 | 2 | 3 | 2 | 192 | 21.7 | 9.48 | 297552.0625 | 2122172.303 |
| A0A0B6VMB2 | 38 | 12 | 33 | 3 | 460 | 50.7 | 7.77 | 31176831.78 | 220474998.3 |
| Q9D051 | 10 | 3 | 4 | 3 | 359 | 38.9 | 6.87 | 4729021.125 | 33428589.13 |
| P11499 | 10 | 7 | 9 | 3 | 724 | 83.2 | 5.03 | 1439819.125 | 9989945.013 |
| P45952 | 17 | 7 | 8 | 7 | 421 | 46.5 | 8.37 | 3358465.75 | 19835954.7 |
| Q9WUM5 | 13 | 4 | 6 | 4 | 346 | 36.1 | 9.39 | 5349782 | 31376325.46 |
| P68040 | 6 | 2 | 4 | 2 | 317 | 35.1 | 7.69 | 3637385.25 | 17026808.28 |
| Q8BFR5 | 9 | 4 | 5 | 4 | 452 | 49.5 | 7.56 | 1427679 | 6401812.877 |
| A2A513 | 14 | 10 | 26 | 6 | 561 | 57 | 5.07 | 310513804.1 | 1349224302 |
| Q7TMM9 | 10 | 4 | 8 | 4 | 445 | 49.9 | 4.89 | 852912.75 | 3509437.613 |

Accession: Protein Registration Number; Coverage: Peptide coverage; Unique Peptides: Number of unique peptides; Peptides: Number of peptides; PSMs: Total number of mass spectra matched to peptides; AAs: Number of amino acids; MWkDa: Molecular weight; Calc.pl: Isoelectric point; Abundances: Relative protein expression.

**Table S6** The antibodies used in this study

| Target antigen | Vendor or Source | Catalog # | Working concentration |
| --- | --- | --- | --- |
| Cardiac Troponin T | Abcam | ab91605 | 1/300 |
| SENP1 | Abcam | Ab108981 | 1/200 for IF; 1/1000 for WB. |
| Alpha smooth muscle Actin | Abcam | ab7817 | 1/200 for IF; 1/1000 for WB. |
| Periostin | Abcam | ab14041 | 1/1000 |
| Vimentin | Abcam | Ab8978 | 1/1000 |
| STAT3 | Abcam | ab109085 | 1/1000 |
| STAT3(phospho Y705) | Abcam | ab76315 | 1/200 for IF; 1/1000 for WB. |
| FAK | Abcam | ab40794 | 1/1000 |
| FAK(phospho Y397) | Abcam | ab81298 | 1/1000 |
| PDGFRB | Abcam | ab32570 | 1/1000 |
| PDGFRB(phospho Y751) | Abcam | ab218534 | 1/1000 |
| VEGFR2 | Abcam | ab221679 | 1/1000 |
| Hsp90 beta | Abcam | ab203085 | 1/200 for IF; 1/1000 for WB. |
| SUMO1 | Abcam | ab11672 | 1/20 for IP; 1/1000 for WB. |
| Lamin B1 | Abcam | ab16048 | 1/800 |
| GAPDH | Abcam | ab9485 | 1/2000 |
| beta Actin | Abcam | ab6276 | 1/2000 |
| Goat anti-rabbit IgG H&L (HRP) | Abcam | ab6712 | 1/20000 |
| Goat anti-mouse IgG H&L (HRP) | Abcam | ab205719 | 1/20000 |
| Goat anti-rabbit IgG H&L (DyLight® 488) | Abcam | ab150077 | 1/500 |
| Goat anti-mouse IgG H&L (DyLight® 594) | Abcam | ab150116 | 1/500 |
| Goat anti-mouse IgG H&L (DyLight® 488) | Abcam | ab150117 | 1/500 |
| Goat anti-rabbit IgG H&L (DyLight® 594) | Abcam | ab150084 | 1/500 |
| DYKDDDDK Tag | Cell Signaling Technology | 2368 | 1/20 for IP; 1/1000 for WB. |
| HA-Tag | Cell Signaling Technology | 3724 | 1/20 for IP; 1/1000 for WB. |
| His-Tag | Cell Signaling Technology | 12698 | 1/20 for IP; 1/1000 for WB. |
| Myc-Tag | Cell Signaling Technology | 2272 | 1/1000 |
| p-VEGFR2 | Cell Signaling Technology | 2478 | 1/1000 |
| Fibronectin | Proteintech | 15613-1-AP | 1/200 for IF; 1/1000 for WB. |
| Alpha Actin | Proteintech | 23660-1-AP | 1/200 |
| Collagen Type I | Proteintech | 14695-1-AP | 1/200 |
